# ^177^Lu-Labeled Iron Oxide Nanoparticles Functionalized with Doxorubicin and Bevacizumab as Nanobrachytherapy Agents against Breast Cancer

**DOI:** 10.3390/molecules29051030

**Published:** 2024-02-27

**Authors:** Evangelia-Alexandra Salvanou, Argiris Kolokithas-Ntoukas, Danai Prokopiou, Maria Theodosiou, Eleni Efthimiadou, Przemysław Koźmiński, Stavros Xanthopoulos, Konstantinos Avgoustakis, Penelope Bouziotis

**Affiliations:** 1 Institute of Nuclear & Radiological Sciences & Technology, Energy & Safety, National Center for Scientific Research “Demokritos”, 15341 Athens, Greece; salvanou@rrp.demokritos.gr (E.-A.S.); staxan@rrp.demokritos.gr (S.X.); 2 Department of Pharmacy, School of Health Sciences, University of Patras, 26504 Patras, Greece; kolokithas@upatras.gr (A.K.-N.); avgoust@upatras.gr (K.A.); 3 Laboratory of Inorganic Chemistry, Department of Chemistry, National and Kapodistrian University of Athens, Panepistimiopolis-Zografou, 15771 Athens, Greece; dprokop@chem.uoa.gr (D.P.); mtheodoss@chem.uoa.gr (M.T.); efthim@chem.uoa.gr (E.E.); 4 Centre of Radiochemistry and Nuclear Chemistry, Institute of Nuclear Chemistry and Technology, Dorodna 16 Str., 03-195 Warsaw, Poland; p.kozminski@ichtj.waw.pl

**Keywords:** Lutetium-177, nanobrachytherapy, iron oxide nanoparticles, MTT, radiolabeling, doxorubicin, bevacizumab, breast cancer, biodistribution, therapeutic efficacy

## Abstract

The use of conventional methods for the treatment of cancer, such as chemotherapy or radiotherapy, and approaches such as brachytherapy in conjunction with the unique properties of nanoparticles could enable the development of novel theranostic agents. The aim of our current study was to evaluate the potential of iron oxide nanoparticles, coated with alginic acid and polyethylene glycol, functionalized with the chemotherapeutic agent doxorubicin and the monoclonal antibody bevacizumab, to serve as a nanoradiopharmaceutical agent against breast cancer. Direct radiolabeling with the therapeutic isotope Lutetium-177 (^177^Lu) resulted in an additional therapeutic effect. Functionalization was accomplished at high percentages and radiolabeling was robust. The high cytotoxic effect of our radiolabeled and non-radiolabeled nanostructures was proven in vitro against five different breast cancer cell lines. The ex vivo biodistribution in tumor-bearing mice was investigated with three different ways of administration. The intratumoral administration of our functionalized radionanoconjugates showed high tumor accumulation and retention at the tumor site. Finally, our therapeutic efficacy study performed over a 50-day period against an aggressive triple-negative breast cancer cell line (4T1) demonstrated enhanced tumor growth retention, thus identifying the developed nanoparticles as a promising nanobrachytherapy agent against breast cancer.

## 1. Introduction

Radiation therapy (RT) is a standard procedure included in cancer treatment protocols. It exploits the intrinsic properties of therapeutic radionuclides, which can be alpha emitters like Actinium-225 (^225^Ac) or beta emitters like Lutetium-177 (^177^Lu). Although ^177^Lu is still considered a “novel” non-conventional radionuclide, there is increasing interest in including it in cancer treatment in the next few years [1]. The effectiveness of ^177^Lu radiopharmaceuticals has also been proven by the fact that Lutathera^®^ and, more recently, Pluvicto^®^ have been approved for the treatment of somatostatin-positive neuroendocrine and prostate tumors, respectively [2]. Most importantly, the beta particles emitted have a maximum energy of 497 keV, allowing for ideal penetration of soft tissue. Furthermore, the convenient half-life of ^177^Lu (t_1/2_ = 6.7 days) offers a longer shelf-life and is ideal for logistical purposes, allowing for the production, preparation, distribution, and therefore availability of the radiopharmaceutical on a much wider basis. Its long half-life also provides prolonged irradiation of the tumor, enhancing the therapeutic potential of the administered therapeutic agent [3]. In RT, dosimetry is an important aspect and gamma emissions from ^177^Lu allow us to conduct single-photon emission tomography (SPECT). Furthermore, the decay of ^177^Lu to stable Hafnium-177 (^177^Hf) makes waste management and patient safety much simpler. However, although therapy with ^177^Lu can be very efficacious against solid tumors, radiation-induced toxicity in healthy tissues and organs is of major concern. Therefore, the targeted delivery of ^177^Lu-labeled radiopharmaceuticals directly to the site of interest is critical and can be achieved with the brachytherapy approach.

Brachytherapy is identified as internal RT and involves the placement of a radioactive source inside or near the tumor [4]. For example, breast brachytherapy has been studied as a supporting treatment as well as a standalone treatment when used in high doses [5,6]. While brachytherapy is a well-established method used in clinical practice, nanobrachytherapy or “nanoscale brachytherapy” is a relatively unexplored field with scarce studies over the last 15 years but with an exponentially increasing amount of interest in the last couple of years [7]. Nanobrachytherapy includes the use of nanoparticles (NPs) instead of seeds or devices (i.e., catheters) [8]. The superiority of nanobrachytherapy is based on the fact that it is a non-invasive technique requiring a simple injection and not a surgical procedure, with no requirement for seed/device removal and therefore no limitation in terms of patient burden, in contrast to conventional brachytherapy techniques.

Nanoparticles have proven to be useful in numerous fields as well as in the medical field as operating at the nanoscale allows for more precise targeting of specific cells or tissues [9]. During the last decade, nanoparticles radiolabeled with diagnostic or therapeutic isotopes have shown great potential in overcoming drawbacks that could not be surpassed with conventional methods, offering the potential for a more personalized cancer treatment approach [10]. Iron oxide and gold nanoparticles are used in diagnostic applications such as magnetic resonance imaging (MRI), self-tests for COVID-19, and recently as potential nanobrachytherapy agents [8,11,12,13]. In particular, iron oxide nanoparticles (IONs) have many unique properties which can be also tuned during their synthesis and functionalization. The functionalization of IONs with targeting moieties and loading with chemotherapeutics could give them the capacity for targeted cancer therapy [14,15,16,17]. Finally, nanoparticles radiolabeled with ^177^Lu either via a chelator or a chelator-free approach have been evaluated concerning their therapeutic potential against cancer [18].

Within this scope, in the present work, we focused on iron oxide nanoparticles, coated with alginic acid and stabilized by polyethylene glycol (MAPEG). Further functionalization of MAPEG with a chemotherapeutic agent (doxorubicin) and with the monoclonal antibody bevacizumab (MAPAD) was performed. The conjugation efficiency was determined by high-performance liquid chromatography (HPLC) and a thermogravimetric analysis (TGA) and the basic physicochemical characteristics by dynamic light scattering (DLS). Afterwards, we performed direct radiolabeling with ^177^Lu. The cytotoxic profile of the different conjugates was evaluated on five different breast cancer cell lines. The cellular uptake of the antibody-functionalized NPs was visualized by fluorescence microscopy and Prussian blue staining. The ex vivo biodistribution of [^177^Lu]Lu-MAPAD was evaluated in 4T1 tumor-bearing mice with three different routes of administration: intravenous, intraperitoneal, and intratumoral. Intratumoral injection showed the optimal biodistribution results, enhancing the local administration of the radionanoconjugate due to the presence of the antibody. The therapeutic effect of [^177^Lu]Lu-MAPAD was assessed in 4T1 xenografts after a single intratumoral injection.

## 2. Results

### 2.1. Functionalization with Doxorubicin and Bevacizumab

**Figure 1:**
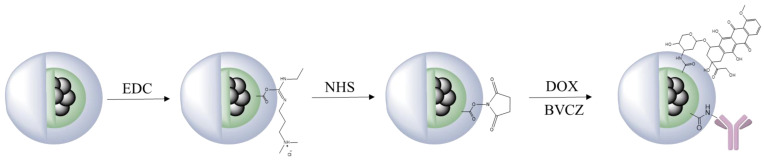
Reaction scheme of the functionalization of MAPEG with DOX and BVCZ.

For the functionalization of MAPEG with bevacizumab (BVCZ) and doxorubicin (DOX), we took advantage of the available carboxyl groups (-COOH) of the alginic acid using N-ethyl-N′-(3-(dimethylamino)propyl)carbodiimide (EDC) and N-hydroxysuccinimide (NHS) crosslinking agents at physiologic pH. The crosslinking reaction scheme is presented in Figure 1.

#### 2.1.1. Antibody Conjugation

**Figure 2:**
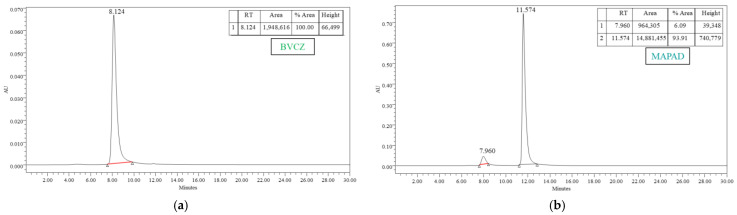
(**a**) Representative HPLC graph of BVCZ (reference sample); (**b**) Representative HPLC graph of unreacted BVCZ.

In order to verify and quantify the antibody conjugation onto the NPs, a high-performance liquid chromatography (HPLC) analysis was performed. As shown in Figure 2a, the characteristic peak of BVCZ is at ~8 min. The reference sample had the total quantity of the antibody added to our reaction (1 mg) and was diluted to a 1 mL volume, as was our reaction sample.

The supernatant of the final step of the functionalization was removed and injected into the HPLC. The non-conjugated amount of BVCZ was calculated by comparing the area under the curve of the unreacted antibody (Figure 2b) to the area under the curve of the reference sample (Figure 2a). Therefore, the binding and entrapment efficiency were calculated by Equations (1) and (2) as follows:

(1) $$\%\;\mathrm{BE}=\left(\frac{359.7\;\mu g\;\mathrm{BVCZ}}{500\;\mu g\;\mathrm{MAPAD}\;+359.7\;\mu g\;\mathrm{BVCZ}}\right)\;\times \;100\%\;=\;41.84\;\mathrm{wt}.\%$$

(2) $$\%\;\mathrm{EE}=\left(\frac{359.7\;\mu g\;\mathrm{BVCZ}}{1000\;\mu g\;\mathrm{BVCZ}}\right)\;\times \;100\%\;=\;35.97\;\mathrm{wt}.\%$$

The functionalization of MAPEG NPs with the antibody was found to be 41.84 wt.%. The second, much bigger peak present in the graph of the unreacted antibody (Figure 2b) comes from the unreacted NHS, which remained in the solution and was removed in the final centrifugation. Additional centrifugations were performed, and the supernatants were analyzed with HPLC in order to verify the total removal of unreacted substances.

#### 2.1.2. Doxorubicin Loading

**Figure 3:**
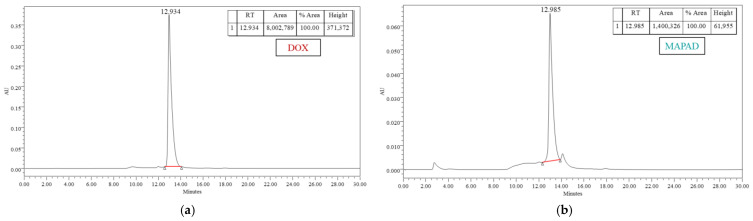
(**a**) Representative RP-HPLC graph of DOX (reference sample); (**b**) Representative HPLC graph of unreacted DOX.

The amount of bound DOX was quantified with reverse-phase HPLC (RP-HPLC). DOX has a very characteristic peak at around 12.9 min as shown in Figure 3a. The reference peak resulted after injecting a concentration of 0.5 mg/mL, which is equal to the initial amount of DOX added to the reaction sample.

As in the case of BVCZ, the area under the curve of the unreacted DOX (Figure 3b) was compared to the respective area under the curve of the reference sample (Figure 3a).

(3) $$\%\;\mathrm{DL}=\left(\frac{413.5\;\mu g\;\mathrm{DOX}}{500\;\mu g\;\mathrm{MAPAD}\;+413.5\;\mu g\;\mathrm{DOX}}\right)\;\times \;100\%\;=\;45.27\;\mathrm{wt}.\%$$

A high percentage of the drug was loaded onto the nanoparticles, reaching almost 50%.

(4) $$\%\;\mathrm{EE}=\left(\frac{413.5\;\mu g\;\mathrm{DOX}}{500\;\mu g\;\mathrm{DOX}}\right)\;\times \;100\%\;=\;82.70\;\mathrm{wt}.\%$$

The above equations show a significant level of DOX entrapment efficiency in the nanoparticles, reaching 82.67 ± 3.87 wt.%. The functionalization of MAPEG NPs only with DOX without the addition of the antibody showed a slightly higher efficiency than that when both BVCZ and DOX were added (88.99% vs. 82.70%).

#### 2.1.3. Thermogravimetric Analysis

**Figure 4:**
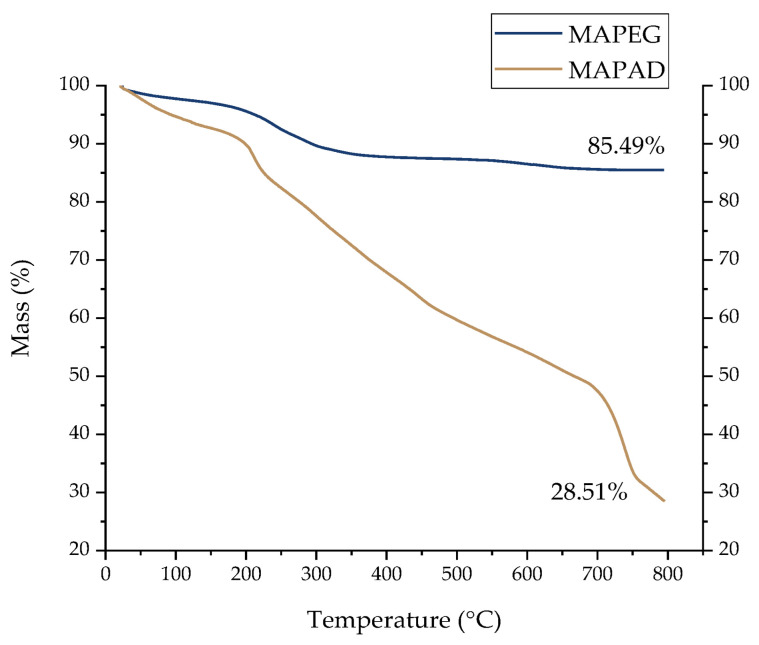
Thermogravimetric analysis graph of MAPEG (blue line) and MAPAD (beige line).

The thermograms demonstrated in Figure 4 indicate a loss of mass. In the case of the pegylated NPs (sample mass of 1.511 mg), the slight weight loss of about 3% observed between 30 and 150 °C is related to the adsorbed free water molecules (free water evaporation). The second loss of mass (8.7%), recorded from 150 °C to 350 °C, is attributed to alginate and PEG degradation. At the most elevated temperatures (350–800 °C), the loss of mass is negligible.

The recorded curve of the MAPAD NPs (sample mass of 0.626 mg) shows the biggest total weight loss, which is around 71.5%. In the thermogram of the NPs decorated with DOX and BVCZ, the first weight loss of about 7.3% can be attributed to moisture (30–150 °C). In the temperature range between 150 °C and 350 °C, we observed a weight loss of ~20%, which can be attributed to alginate and PEG degradation (8.7%, as shown for MAPEG), and another 11.4% mass loss due to antibody or drug degradation. The last weight loss from 400 to 800 °C reached 44.0% and may be attributed to the destruction of DOX and BVCZ.

#### 2.1.4. Dynamic Light Scattering

The hydrodynamic diameter (D_h_) and zeta potential (ζ_p_) of the newly synthesized NPs were determined using DLS. The hydrodynamic diameter as well as the zeta potential were found to be 474.6 ± 13.8 nm and 16.7, respectively.

**Figure 5:**
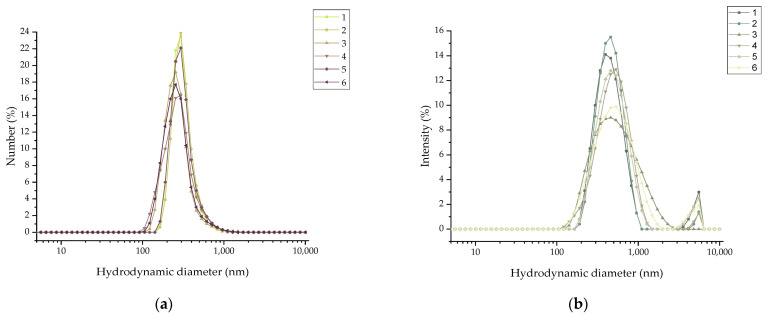
DLS graphs of size distribution of MAPAD against (**a**) number and (**b**) intensity.

As presented in the figure above, there are some differences between the size distribution against the number (Figure 5a) and intensity (%) (Figure 5b), which can be explained by all the phenomena and interactions taking place among the different functionalities and water molecules or even among different particles. The much larger peaks in the graph of the size distribution against intensity are most likely due to the presence of dust particles in the sample.

### 2.2. DOX Release

**Figure 6:**
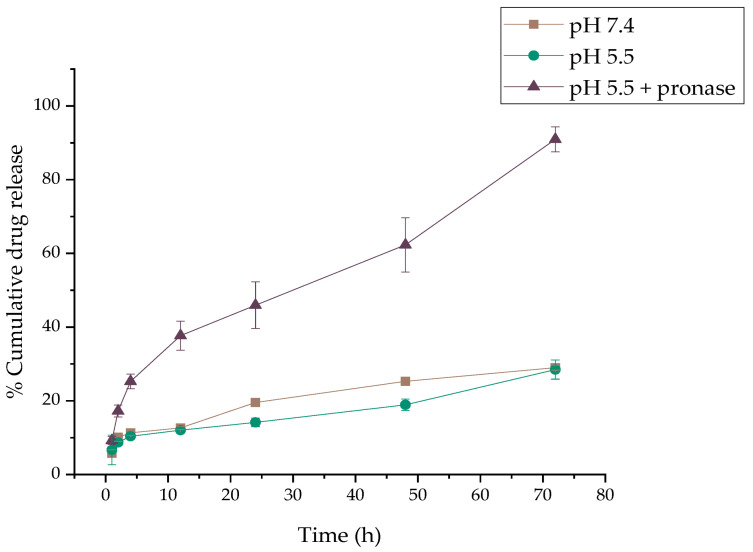
Cumulative release of DOX in PB at a pH of 7.4 (brown line), PB at a pH of 5.5 (green line), and PB at a pH of 5.5 enriched with pronase (purple line).

The release profiles of DOX from the functionalized NPs’ MAPAD, which was determined in vitro in phosphate buffer (PB) at a pH of 7.4 and an acidic pH (PB pH of 5.5) with and without pronase, an enzyme which hydrolyzes the amide bonds that connect the DOX molecules to the NPs, are presented in Figure 6. The release of the entrapped DOX was very weak both in PB at pHs of 7.4 and 5.5 in the first hour (5.75 ± 0.93% and 6.69 ± 4.00%), which showed a small increase up to 12 h (12.65 ± 0.42% and 12.06 ± 0.73%, respectively). More than 70.99% and 71.51% of the encapsulated DOX was retained on the MAPAD after 72 h of incubation at 37 °C. In contrast, in PB at a pH of 5.5 enriched with pronase, the DOX release was enhanced (37.68 ± 3.94% at 12 h). After 24 h of incubation with pronase, a cumulative release of 45.93 ± 6.32% was observed, which increased to 62.32 ± 7.38% at 48 h and reached 90.94 ± 3.40% after 72 h. Consequently, the presence of pronase substantially accelerated the release of DOX from MAPAD.

### 2.3. Radiolabeling with ^177^Lu

The radiolabeling of the pegylated nanoparticles (MAPEG) with ^177^Lu has been evaluated and described previously [19]. Briefly, [^177^Lu]Lu-MAPEG occurred after the incubation of the MAPEG NPs with ^177^Lu for 30 min at 75 °C. The radiochemical yield reached 93.65 ± 1.03% and the radiolabeled nanoconjugate was stable up to 7 days post preparation (95.60 ± 2.03%), also demonstrating a serum stability ([^177^Lu]Lu-MAPEG:serum 1:10 *v*/*v*, at 37 °C) ranging from 77.29 ± 1.69% to 70.72 ± 1.75% at 2 h and 7 d post radiolabeling, respectively.

**Figure 7:**
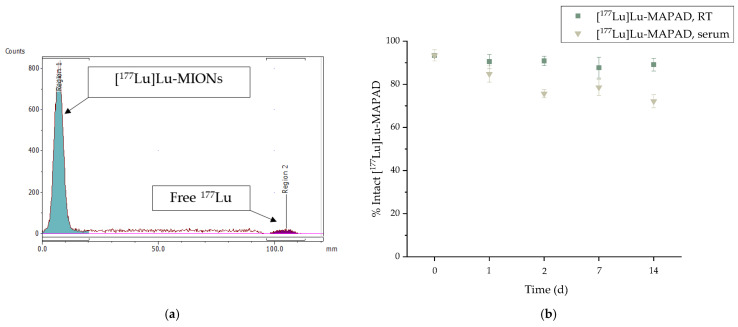
(**a**) Representative radio-TLC graph of ^177^Lu-labeled MIONs; (**b**) Radiochemical stability of [^177^Lu]Lu-MAPAD at RT and in human serum (x axis not in scale).

For the radiolabeling of the antibody-functionalized NPs (MAPAD) with ^177^Lu, the temperature was kept lower so as not to compromise the antibody immunoreactivity. After overnight incubation at 40 °C, direct radiolabeling with ^177^Lu was successful with radiolabeling yields reaching 93.41 ± 2.59%. The assessment of the radiochemical yield and stability was performed with radio-TLC, as represented in Figure 7a.

The in vitro stability of [^177^Lu]Lu-MAPAD was evaluated up to 14 d post radiolabeling (Figure 7b). The ^177^Lu-labeled NPs were assessed regarding their stability at RT as well as at serum at a ratio of 1:10 *v*/*v* ([^177^Lu]Lu-MAPAD:serum) and 37 °C. The percentage of intact [^177^Lu]Lu-MAPAD at RT was >80% (89.12 ± 2.99%) even at 14 d post radiolabeling. Additionally, the serum stability ranged from 84.80 ± 3.75% to 72.16 ± 3.05% at 1 d and 14 d post radiolabeling, respectively.

### 2.4. In Vitro Cytotoxicity

#### 2.4.1. In Vitro Cytotoxicity of MAPEG

**Figure 8:**
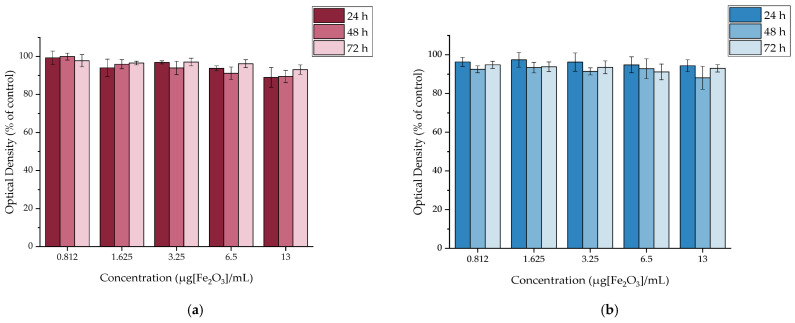

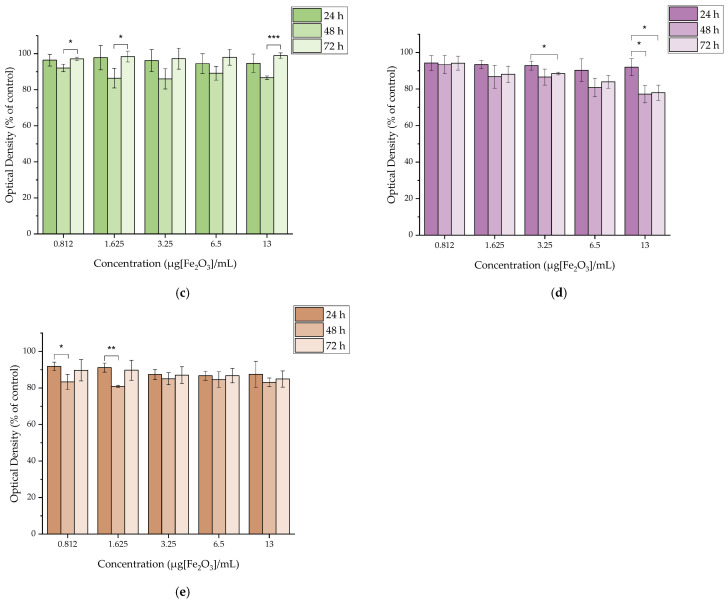
MTT assay of MAPEG after 24, 48, and 72 h against: (**a**) 4T1; (**b**) MDA-MB-231; (**c**) M165; (**d**) MCF7; (**e**) SKBR3 cell lines. Mean values (*n* = 3) and the SD (bars) are shown (x axis not in scale). Asterisks indicate the statistical significance of the difference between the results (* *p* < 0.05, ** *p* < 0.01, *** *p* < 0.001). Absence of asterisks denotes a non-significant statistical difference.

The cytotoxicity of MAPEG NPs was evaluated against the 4T1, MDA-MB-231, M165, MCF7, and SKBR3 cell lines as demonstrated in Figure 8. The concentrations ranged from 0.8125 to 13 μg[Fe_2_O_3_]/mL. It is obvious that in all cases, the cell viability does not drop below 70% even after 72 h of treatment, which suggests the pegylated nanoparticles have a good level of biocompatibility.

#### 2.4.2. In Vitro Cytotoxicity of [^177^Lu]Lu-MAPEG

**Figure 9:**
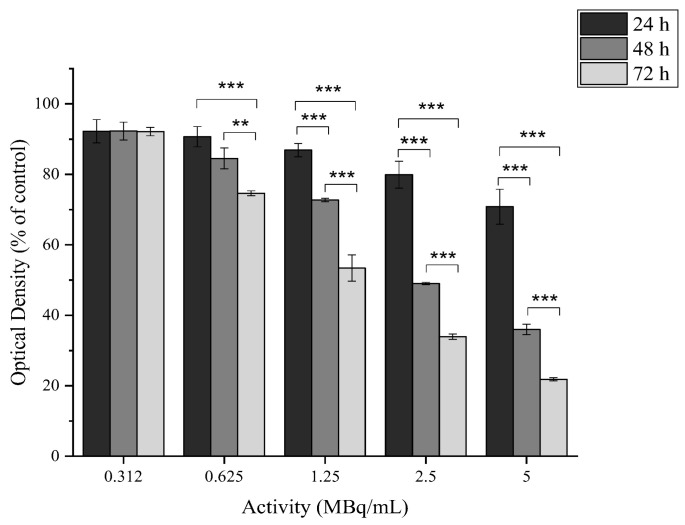
MTT assay of [^177^Lu]Lu-MAPEG against the 4T1 cell line after 24 h. Mean values (*n* = 3) and the SD (bars) are shown (x axis not in scale). Asterisks indicate the statistical significance of the difference between the results (** *p* < 0.01, *** *p* < 0.001). Absence of asterisks denotes a non-significant statistical difference.

The cytotoxic effect of the ^177^Lu-labeled MAPEG nanoparticles was evaluated in 4T1 breast cancer cells at 24, 48, and 72 h. The radioactivity ranged between 0.312 and 5 MBq/mL, which corresponds to the non-radiolabeled MAPEG concentrations of 0.8125 to 13 μg[Fe_2_O_3_]/mL (Figure 9).

After 24 h of incubation with [^177^Lu]Lu-MAPEG, the highest difference was noted for the 5 MBq/mL sample, for which the cell viability was 70.82 ± 4.96% versus 89.00 ± 5.20% for the non-radiolabeled MAPEG, denoting statistical significance (* *p* = 0.0119). The different effect of the radiolabeled and non-radiolabeled NPs is also obvious after 72 h of treatment, at which the 1.625 μg[Fe_2_O_3_]/mL viability is 96.52 ± 0.99% versus 74.61 ± 0.71% (*** *p* < 0.0001). As the activity is increased, this difference increases significantly (93.04 ± 2.49 vs. 21.84 ± 0.47%, *** *p* < 0.0001). Therefore, this difference in cell viability indicates a dose-dependent toxicity attributed to the presence of ^177^Lu.

#### 2.4.3. In Vitro Cytotoxicity of MAPAD

**Figure 10:**
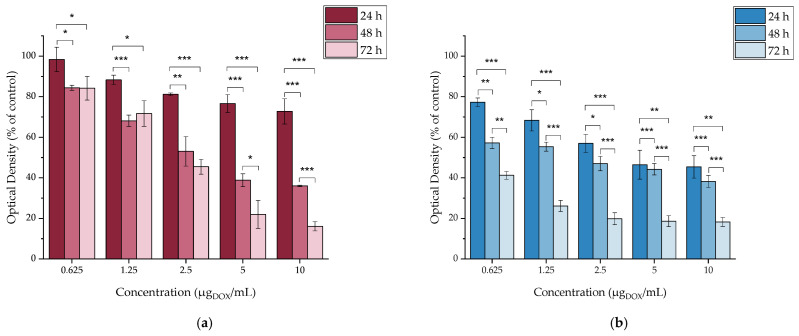

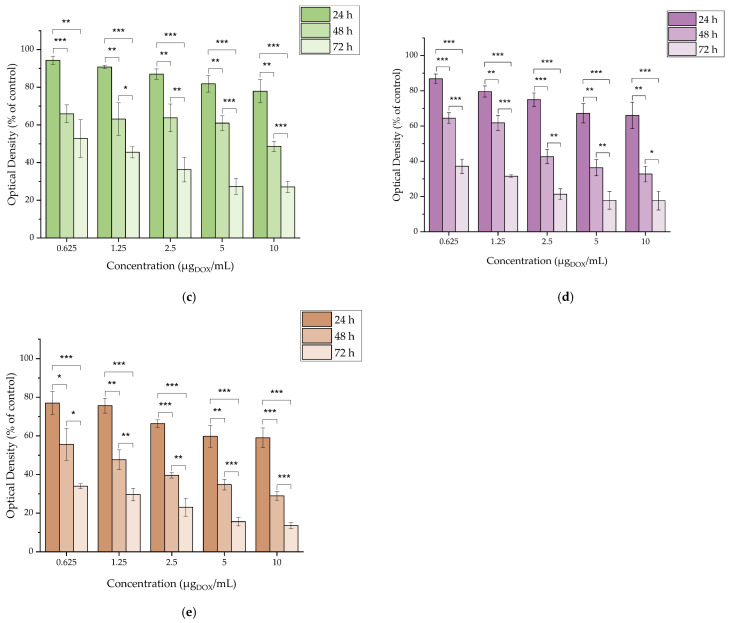
MTT assay of MAPAD after 24, 48, and 72 h against: (**a**) 4T1; (**b**) MDA-MB-231; (**c**) M165; (**d**) MCF7; (**e**) SKBR3 cell lines. Mean values (*n* = 3) and the SD (bars) are shown (x axis not in scale). Asterisks indicate the statistical significance of the difference between the results (* *p* < 0.05, ** *p* < 0.01, *** *p* < 0.001). Absence of asterisks denotes a non-significant statistical difference.

The toxicity of the functionalized MAPAD NPs was evaluated against all cancer cell lines (4T1, MDA-MB-231, M165, MCF7, and SKBR3) at 24, 48, and 72 h. In this case, the concentrations were determined based on the quantity of the cytotoxic drug (DOX). The examined range of DOX concentrations was from 0.625 up to 10 μg_DOX_/mL, and the results are presented in Figure 10.

#### 2.4.4. In Vitro Cytotoxicity of DOX

**Figure 11:**
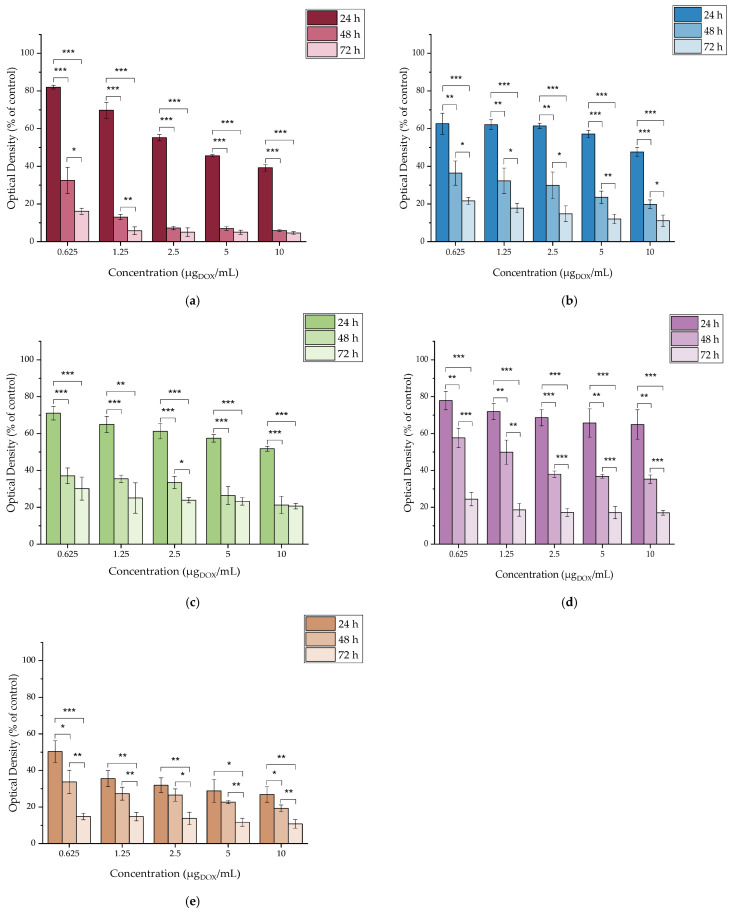
MTT assay of DOX after 24, 48, and 72 h against: (**a**) 4T1; (**b**) MDA-MB-231; (**c**) M165; (**d**) MCF7; (**e**) SKBR3 cell lines. Mean values (*n* = 3) and the SD (bars) are shown (x axis not in scale). Asterisks indicate the statistical significance of the difference between the results (* *p* < 0.05, ** *p* < 0.01, *** *p* < 0.001). Absence of asterisks denotes a non-significant statistical difference.

The cytotoxicity of DOX up to 72 h was assessed in all cancer cell lines in order to have a clear view of the cytotoxicity of each nanoparticle component separately. DOX is highly cytotoxic against all of the investigated cell lines, as demonstrated in Figure 11. Our results showed that even at the lowest concentration tested, after 24 h of treatment, the cell viability of all cell lines was <85%. However, it is worth noting that each cancer cell line tested had a different response to treatment with the drug.

The most significant responses were from SKBR3 cells, for which after 24 h of incubation with DOX, the cell viability ranged from 50.33 ± 5.99% to 26.85 ± 4.23% at the lowest and highest concentrations, respectively. Another important aspect is the pronounced response of the 4T1 cell line, showing the highest viability (82.04 ± 1.01%) after 24 h of incubation at 0.625 μg_DOX_/mL, which drops to 16.11 ± 1.64% after 72 h of incubation (*** *p* < 0.0001). Additionally, at the same time point, the treatment with the highest DOX concentration was reported to have the lowest viability (4.57 ± 0.72%) among all cell lines.

#### 2.4.5. In Vitro Cytotoxicity of [^177^Lu]LuCl_3_

**Figure 12:**
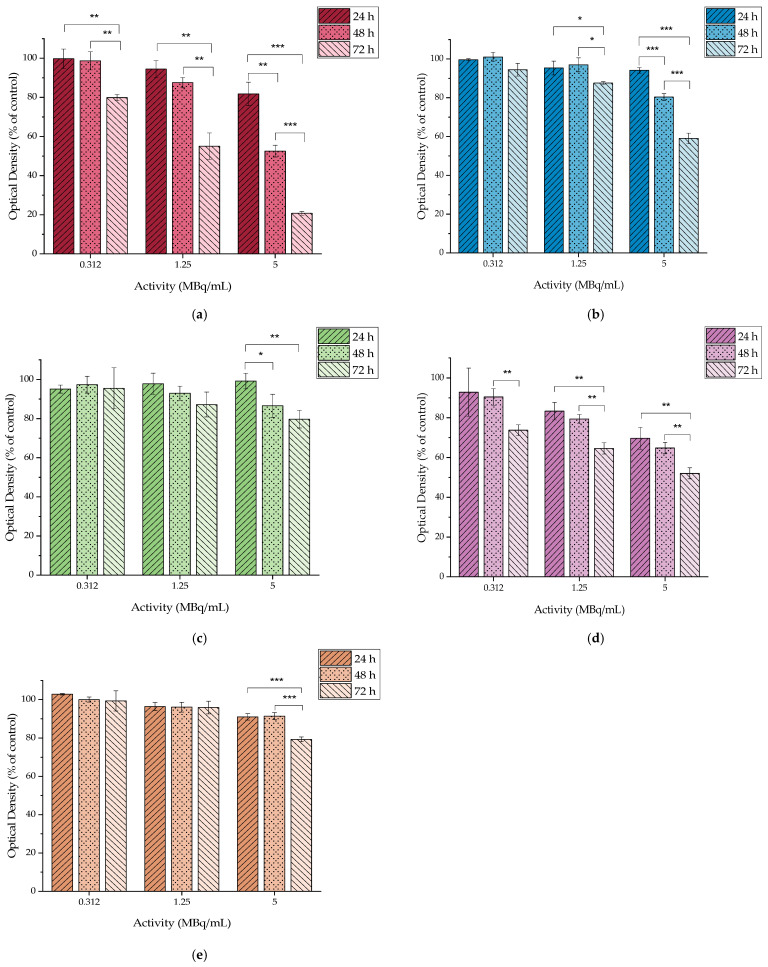
MTT assay of [^177^Lu]LuCl_3_ after 24, 48, and 72 h against: (**a**) 4T1; (**b**) MDA-MB-231; (**c**) M165; (**d**) MCF7; (**e**) SKBR3 cell lines. Mean values (*n* = 3) and the SD (bars) are shown (x axis not in scale). Asterisks indicate the statistical significance of the difference between the results (* *p* < 0.05, ** *p* < 0.01, *** *p* < 0.001). Absence of asterisks denotes a non-significant statistical difference.

The cytotoxic profile of [^177^Lu]LuCl_3_ was assessed in the aforementioned cell lines with 0.312, 1.25, and 5 MBq/mL (Figure 12).

It is impressive how the presence of the radioactive element (^177^Lu) affects the cell viability of 4T1 cells, ranging from 79.86 ± 1.63% (for 0.312 MBq/mL) to 20.75 ± 1.00% (for 5 MBq/mL) at 72 h of incubation, while at the same time, it seems to have a minimal effect on the MDA-MB-231, M165, and SKBR3 cells. MCF7 seems to have an intermediate response to the radioactivity with viability ranging from 73.75 ± 2.70% to 52.02 ± 2.78% after 72 h of incubation.

#### 2.4.6. In Vitro Cytotoxicity of [^177^Lu]Lu-MAPAD

**Figure 13:**
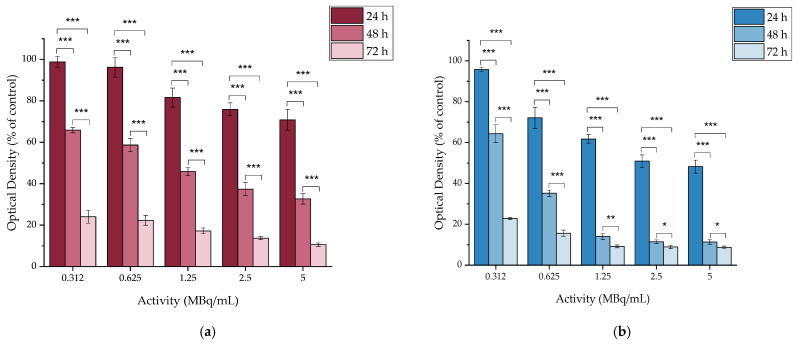

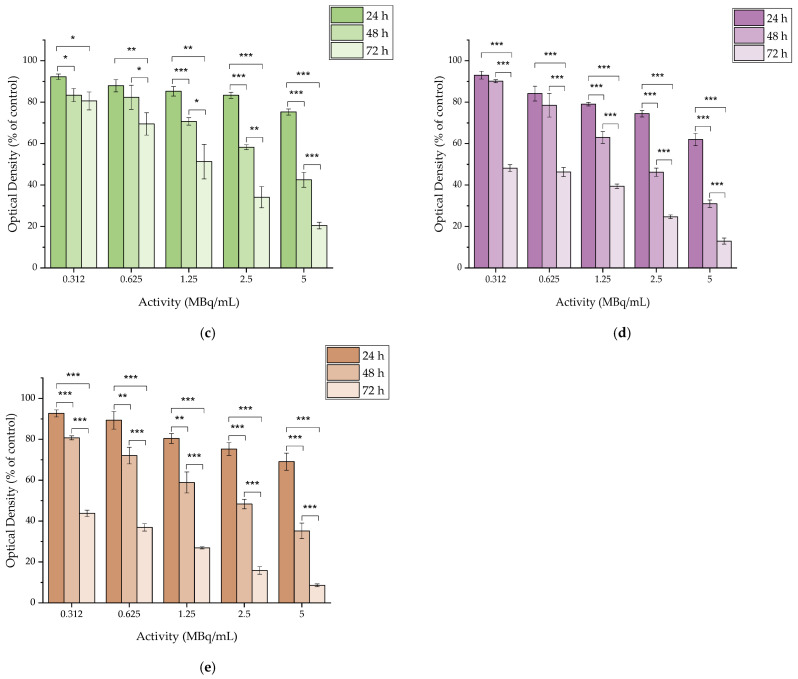
MTT assay of [^177^Lu]Lu-MAPAD after 24, 48, and 72 h against: (**a**) 4T1; (**b**) MDA-MB-231; (**c**) M165; (**d**) MCF7; (**e**) SKBR3 cell lines. Mean values (*n* = 3) and the SD (bars) are shown (x axis not in scale). Asterisks indicate the statistical significance of the difference between the results (* *p* < 0.05, ** *p* < 0.01, *** *p* < 0.001). Absence of asterisks denotes a non-significant statistical difference.

The cytotoxic profile of the [^177^Lu]Lu-MAPAD was evaluated against the same five breast cancer cell lines at 24, 48, and 72 h. The radioactivity ranged between 0.312 and 5 MBq/mL, which, as summarized in Figure 13, corresponds to the non-radiolabeled MAPAD concentrations of 0.8125 to 13 μg[Fe_2_O_3_]/mL and DOX concentrations of 0.625–10 μg/mL.

Figure 13 shows evidence that the ^177^Lu-labeled MAPAD NPs induce a dose- and time-dependent cytotoxicity on all of the tested cell lines. More specifically, the cytotoxic effect is pronounced in both 4T1 and MDA-MB-231, for which at 48 h of incubation with [^177^Lu]Lu-MAPAD, the viability ranges from 65.85 ± 1.29% to 32.67 ± 2.52% and from 64.36 ± 4.36% to 11.33 ± 1.11% for the 4T1 and MDA-MB-231, respectively. The difference between these values and the respective ones for the non-labeled NPs are considered to be statistically significant (4T1 cells: 65.85 ± 1.29% vs. 84.41 ± 1.25%, *** *p* < 0.0001, MDA-MB-231: 11.33 ± 1.11% vs. 38.22 ± 2.94%, *** *p* = 0.0001). This difference increases more at the 72 h time point when the 4T1 cells incubated with the ^177^Lu-labeled MAPAD exhibited a high decrease in cell viability for all tested activities in comparison to that of MAPAD (24.04 ± 3.11% vs. 84.17 ± 5.82% *** *p* < 0.0001, 22.35 ± 2.38% vs. 71.70 ± 6.33% *** *p* = 0.0002, 17.21 ± 1.41% vs. 45.49 ± 3.67% *** *p* = 0.0002, 13.69 ± 0.68% vs. 22.01 ± 6.88% ns and 10.54 ± 0.84% vs. 16.13 ± 2.28% * *p* = 0.0163). The respective percentages for the MDA-MB-231-treated cells were 22.82 ± 0.58% vs. 41.24 ± 1.94% (*** *p* < 0.0001), 15.65 ± 1.51% vs. 26.14 ± 2.73% (** *p* = 0.0043), 9.18 ± 0.70% vs. 19.83 ± 2.93% (** *p* = 0.0036), 8.89 ± 0.88% vs. 18.64 ± 2.71% (** *p* = 0.0041), and 8.72 ± 0.64% vs. 18.27 ± 2.23% (** *p* = 0.0020).

### 2.5. Fluorescence Microscopy

The cellular uptake and intracellular localization of MAPAD in the 4T1 cell line was examined with fluorescence microscopy after treatment with the nanoconjugates for 1 and 4 h. For the fluorescence imaging, we exploited the fluorescent ability of DOX when excited at 470 nm. In order to identify the localization of the tested nanoconjugate, the obtained results were compared to the uptake of the cells when treated with free DOX and with nanoparticles loaded with DOX but without the antibody (MAPEGDOX). The mechanism of action of DOX is based on its entering the cell nucleus and poisoning topoisomerase-II, resulting in DNA damage and cell death [20]. This is also reported in several studies, in which cell nuclei stained with DAPI (4′,6-diamidino-2-phenylindole) show co-localization with DOX in the nuclei [21,22,23].

The incubation of the cells was performed with 10 μg_DOX_/mL as at that concentration, a higher uptake of the free DOX by the cells is observed, which translates into a more intense fluorescent image. Also, the cytotoxic effect of the drug at that concentration is not capable of killing the treated cells within the examined time period [24].

Control images are included to show that the untreated cells (only the medium) do not have any fluorescent abilities. The second column, in which DOX is added to the medium, clearly shows that when the cells are incubated with free DOX, intense fluorescence can be observed in the nucleus [25].

**Figure 14:**
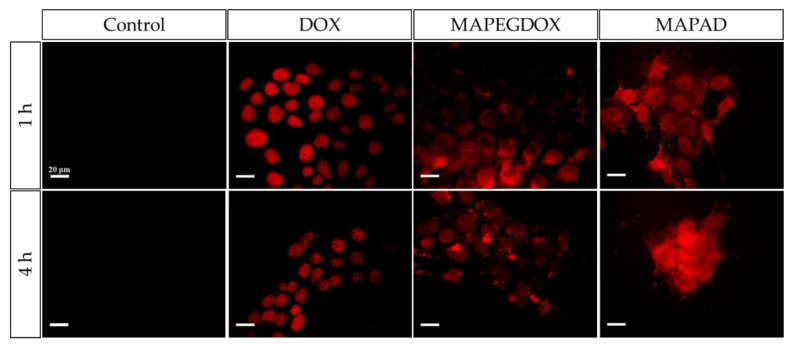
Fluorescence microscopy images of DOX-loaded samples. Images were acquired after 1 h and 4 h of treatment with medium, DOX, MAPEGDOX, and MAPAD of 4T1 cancer cells (scale at 20 μm).

As evidenced in Figure 14, samples of nanoparticles loaded with DOX but without BVCZ (MAPEGDOX) have a tendency to remain in the cytoplasm. Even at 4 h, MAPEGDOX appears to be in the cytoplasm or even outside the cells with a very low signal coming from the nucleus. In contrast, we can see the localization of MAPAD in the cells quite distinctly. In this case, after 1 h of incubation, apart from the nucleus, we can also detect MAPAD NPs in the cytoplasm or even outside the cells, whereas after 4 h of incubation with MAPAD, an intense signal is detected, supporting the successful internalization of our NPs in the 4T1 cells.

### 2.6. Prussian Blue

**Figure 15:**
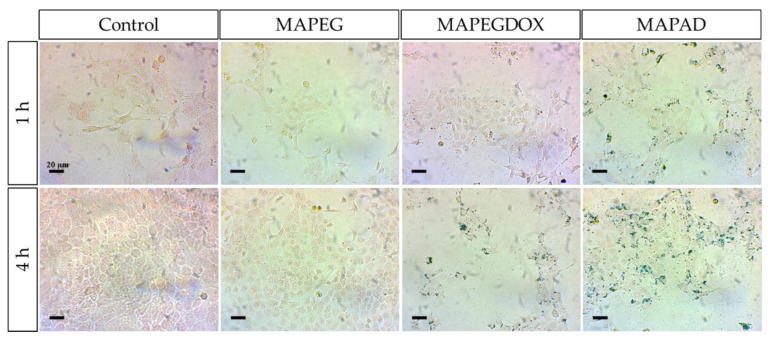
Optical microscopy images stained with Prussian blue. Images were acquired after 1 h and 4 h of treatment with medium, MAPEG, MAPEGDOX, and MAPAD of 4T1 cancer cells (scale at 20 μm).

The MIONs’ cellular uptake was observed after Prussian blue staining using optical microscopy (Figure 15). The Prussian blue staining revealed differences depending on the tested formulation. Our results clearly show that there was no interaction at the examined time points between the pegylated NPs (MAPEG) and 4T1 cells, whereas MAPEGDOX demonstrated a time-dependent increase in cellular uptake. MAPAD NPs, on the other hand, showed uptake in 4T1 cells even after 1 h of incubation. An obvious increase in cellular uptake was observed after 1 and 4 h treatment of the cells with MAPAD, which was substantially augmented when compared to that of MAPEGDOX, respectively.

### 2.7. Ex Vivo Biodistribution

After functionalization with the antibody and the chemotherapeutic, a full investigation in 4T1 tumor-bearing mice was conducted. Intravenous, intraperitoneal, and intratumoral modes of administration were evaluated 1, 2, and 7 d post injection.

#### 2.7.1. Intravenous Administration

**Figure 16:**
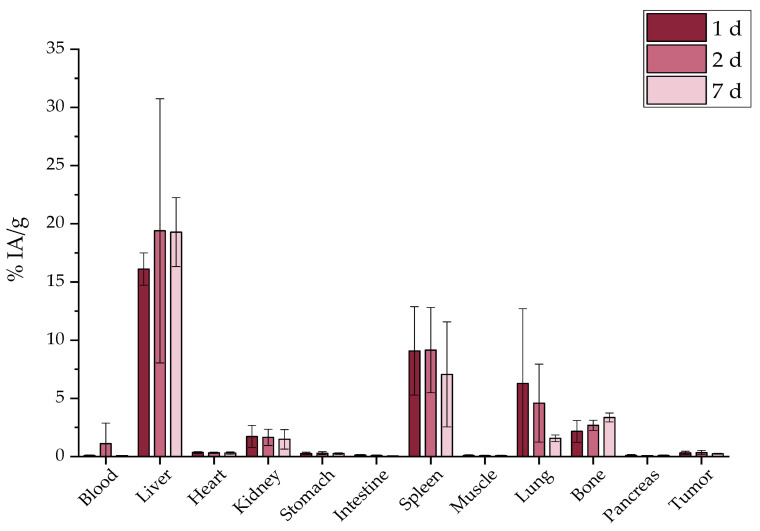
Ex vivo biodistribution after i.v. administration of [^177^Lu]Lu-MAPAD in 4T1 xenografts expressed as % IA/g (*n* = 3).

The intravenous (i.v.) injection of the [^177^Lu]Lu-MAPAD demonstrated an augmented uptake in liver, spleen, and bone at 1 d p.i. (16.10 ± 1.39%, 9.08 ± 3.80%, and 2.16 ± 0.93%, respectively) which remained almost stable up to 7 d p.i. (Figure 16).

#### 2.7.2. Intraperitoneal Administration

**Figure 17:**
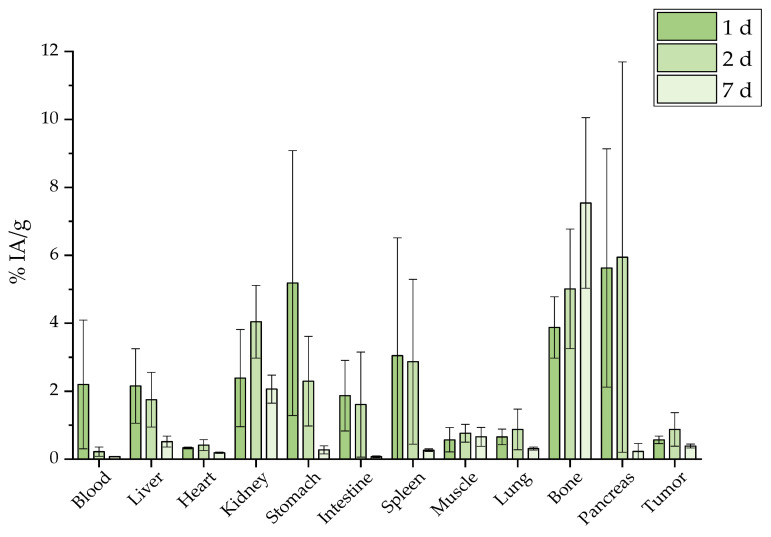
Ex vivo biodistribution after i.p. administration of [^177^Lu]Lu-MAPAD in 4T1 xenografts expressed as % IA/g (*n* = 3).

The intraperitoneal (i.p.) administration (Figure 17) showed a non-specific uptake in most organs which appeared to have an accumulation of less than 5% IA/g, demonstrating an upward trend for bone uptake with the highest percentage noted at 7 d p.i. (7.54 ± 2.51%).

#### 2.7.3. Intratumoral Administration

**Figure 18:**
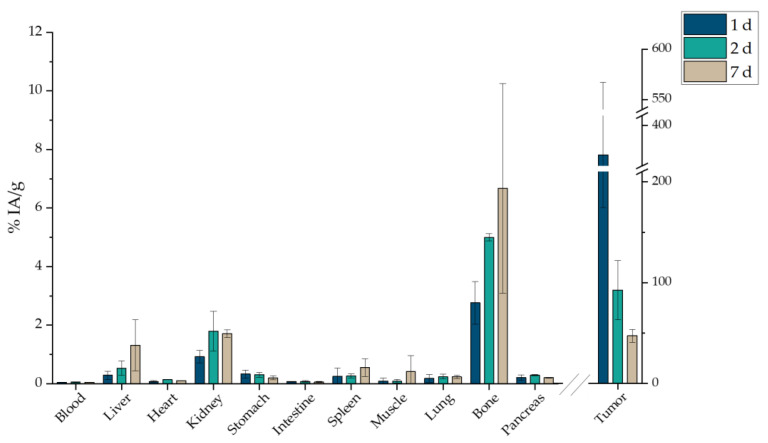
Ex vivo biodistribution after i.t. administration of [^177^Lu]Lu-MAPAD in 4T1 xenografts expressed as % IA/g (*n* = 3).

Since the other two ways of administration did not obtain favorable results, [^177^Lu]Lu-MAPAD was intratumorally (i.t.) administered in 4T1 tumor-bearing xenografts. The mice were euthanized 1, 2, and 7 d post injection, and their biodistribution results are depicted in Figure 18. Indeed, the nanosystem is detected at the tumor site at a pronounced percentage 1 d after the injection (370.69 ± 196.33%), which reached 92.50 ± 29.45% 24 h later. A significant amount is still retained at the tumor even at 7 d p.i. (47.28 ± 6.49%). It is also very important to note that it is not accumulated in any other major organs (liver and kidney < 2% at 7 d p.i.). Nonetheless, a small uptake is noted in bone with an increasing trend, which, however, does not exceed 10% of the IA/g.

**Figure 19:**
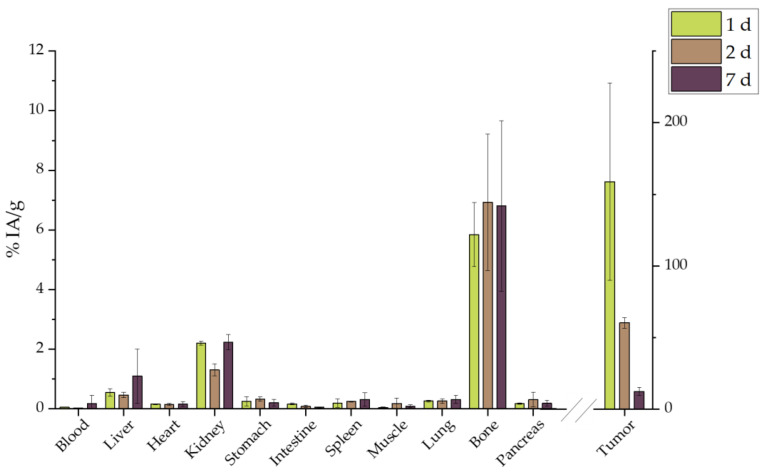
Ex vivo biodistribution after i.t. administration of [^177^Lu]Lu-MAPEG in 4T1 xenografts expressed as % IA/g (*n* = 3).

In order to directly compare whether functionalization with BVCZ actually made a difference to the in vivo behavior of our nanosystem and led to prolonged uptake at the tumor site, the ex vivo biodistribution of intratumorally injected [^177^Lu]Lu-MAPEG in 4T1 xenografts was performed at the same time points (Figure 19). A biodistribution profile similar to that of [^177^Lu]Lu-MAPAD was demonstrated regarding the liver and kidney uptake (0.54 ± 0.13% and 2.20 ± 0.07% at 1 d p.i., respectively), while the bone accumulation ranged from 5.85 ± 1.08% at 1 d p.i. to 6.81 ± 2.86% at 7 d p.i. However, the main difference lies in the tumor accumulation, which was 158.85 ± 68.88 at 1 d p.i., 60.29 ± 3.84% at 2 d, and 12.44 ± 2.71% at 7 d p.i.

**Figure 20:**
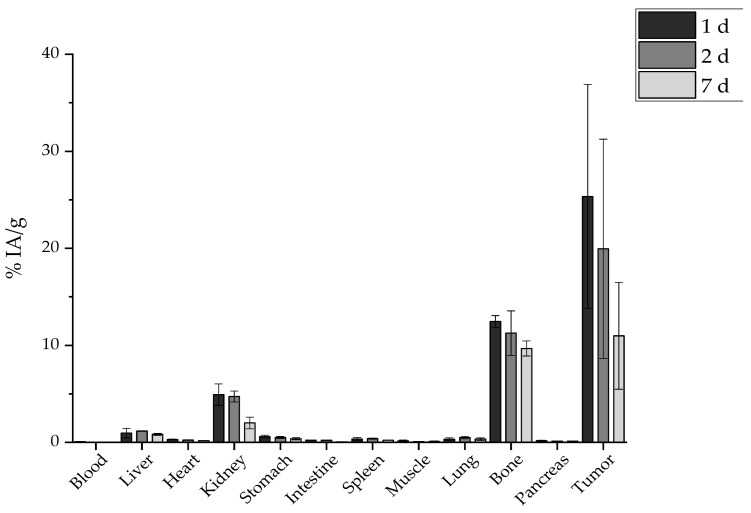
Ex vivo biodistribution after i.t. administration of [^177^Lu]LuCl_3_ in 4T1 xenografts expressed as % IA/g (*n* = 3).

Furthermore, the same direct comparison can be also made with the radioisotope solution ([^177^Lu]LuCl_3_) after i.t. administration in tumor-bearing mice (Figure 20). In this case, there is a distinct difference in the kidney uptake at 1 d p.i. (4.92 ± 1.11% vs. 0.92 ± 0.21%, ** *p* = 0.0036). There is an extremely statistically significant difference considering bone uptake, which in the case of [^177^Lu]LuCl_3_ is 12.46 ± 0.60% (vs. 2.76 ± 0.72%, *** *p* < 0.0001) 1 d after the i.t. administration. Also, the injected substance is not retained at the tumor since only 10.98 ± 5.51% is detected there 1 week later.

**Figure 21:**
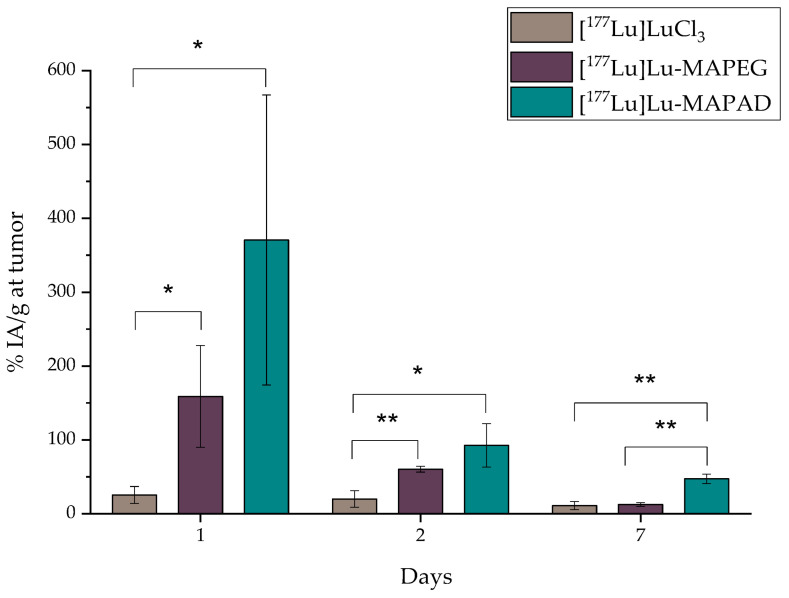
Comparison of the tumor retention after i.t. administration of [^177^Lu]LuCl_3_ vs. [^177^Lu]Lu-MAPEG vs. [^177^Lu]Lu-MAPAD in 4T1 xenografts expressed as % IA/g (*n* = 3). Asterisks indicate the statistical significance of the difference between the results (* *p* < 0.05, ** *p* < 0.01). Absence of asterisks denotes a non-significant statistical difference.

Figure 21 highlights the significantly higher tumor retention of the BVCZ-functionalized nanoparticles in comparison to that of [^177^Lu]LuCl_3_ and [^177^Lu]Lu-MAPEG. The ex vivo biodistribution of [^177^Lu]LuCl_3_ was only performed to facilitate comparisons, as regulations forbid the direct administration of Lutetium chloride.

The biggest difference is noted 1 d post administration with approximately 25.34 ± 11.56%, 158.85 ± 68.88%, and 370.69 ± 196.33% for [^177^Lu]LuCl_3_, [^177^Lu]Lu-MAPEG, and [^177^Lu]Lu-MAPAD, respectively. The nanosystems are drained from the tumor 24 h later, when these percentages drop to 19.95 ± 11.31%, 60.29 ± 3.84%, and 92.50 ± 29.45%. Finally, one week after the administration, almost 10% are still at the tumor site from the injected [^177^Lu]LuCl_3_ and [^177^Lu]Lu-MAPEG (10.98 ± 5.51% and 12.44 ± 2.71%), whereas the corresponding amount of [^177^Lu]Lu-MAPAD retained at the tumor is five times higher (47.28 ± 6.49%). The results clearly demonstrate that functionalization with BVCZ leads to pronounced retention at the tumor site due to the VEGF expression of 4T1 cells. This is not the case for [^177^Lu]Lu-MAPEG or [^177^Lu]LuCl_3_, even though they have also been administered intratumorally, as they do not have a targeting vector that could help them remain at the tumor site. Consequently, the majority of the injected quantity leaks from the tumor at a much faster rate and is distributed in other organs/tissues or eliminated from the body.

### 2.8. Therapeutic Efficacy

**Figure 22:**
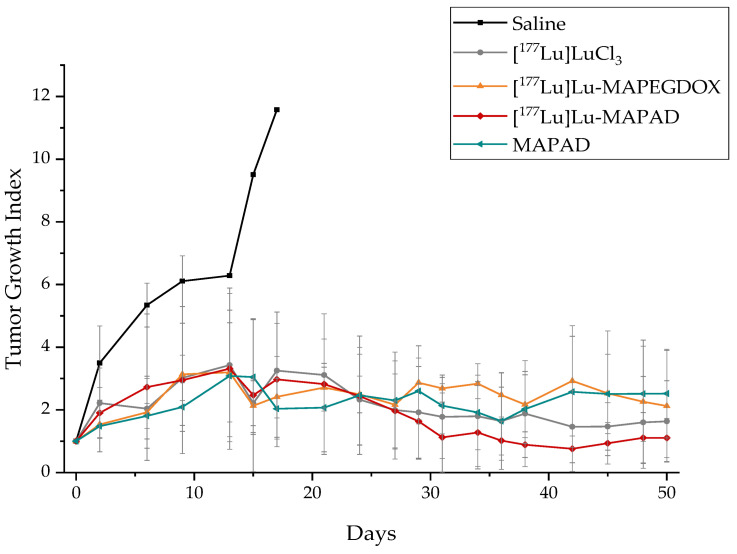
Effect of intratumoral injection of saline (black line), [^177^Lu]LuCl_3_ (gray line), [^177^Lu]Lu-MAPEGDOX (orange line), MAPAD (blue line), and [^177^Lu]Lu-MAPAD (red line) on the tumor growth index (TGI) of 4T1 tumor-bearing mice. Values represent the mean ± SD (*n* = 4 mice per group).

The therapeutic efficacy of [^177^Lu]Lu-MAPAD was determined by an estimation of the tumor growth index (TGI) of five groups of 4T1 tumor-bearing SCID mice, up to 50 days post treatment by a single intratumoral injection. The mice in Group A received one injection of saline (control group: Figure 22, black line), Group B received one injection of [^177^Lu]LuCl_3_ (Figure 22, gray line), and mice in Groups C (Figure 22, orange line) and D (Figure 22, red line) received one injection of ^177^Lu-labeled pegylated nanoparticles functionalized only with doxorubicin (^177^Lu-MAPEGDOX) and ^177^Lu-labeled MAPAD, respectively. The radioactive samples had a radioactivity of ~5 MBq. An extra group (Group E: Figure 22, blue line) received one intratumoral injection of non-radiolabeled MAPAD NPs in order to compare and assess the therapeutic effect of the functionalized nanoparticles without the effect of the radioisotope.

**Figure 23:**
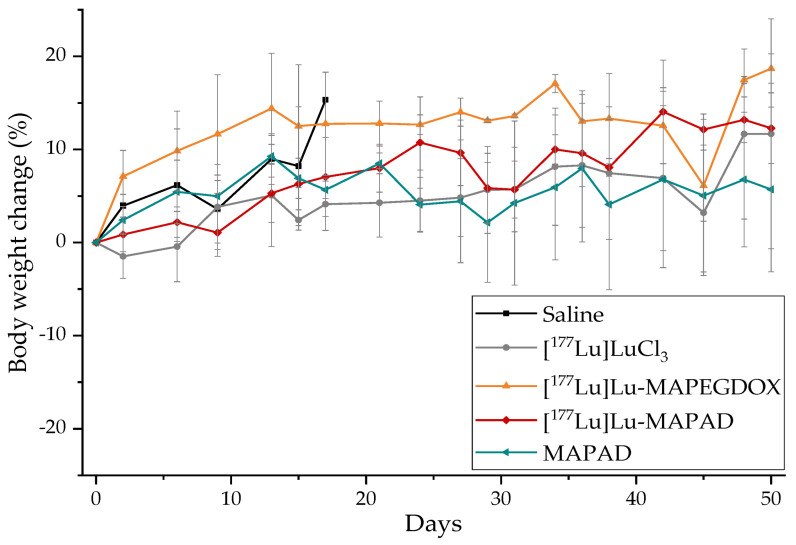
Percentage body weight of mice in the 5 different groups of the therapeutic efficacy study: saline (black line), [^177^Lu]LuCl_3_ (gray line), [^177^Lu]Lu-MAPEGDOX (orange line), MAPAD (blue line), and [^177^Lu]Lu-MAPAD (red line). Values represent the mean change in body weight of each group ± SD (*n* = 4 mice per group).

Loss of body weight is a substantial indicator of toxicity. Therefore, the body weight of the animals included in the therapeutic efficacy study was monitored regularly (three times/week) and is demonstrated in Figure 23. It is obvious that no animal exceeded the humane endpoint criteria (>20% of initial body mass or >15% in 2 days).

**Figure 24:**
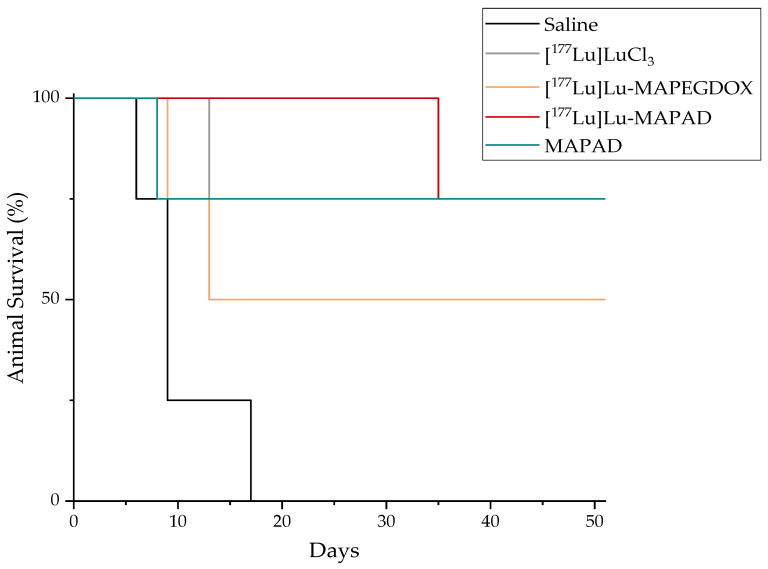
Kaplan–Meier survival curves of 4T1 xenografts: saline (black line), [^177^Lu]LuCl_3_ (gray line), [^177^Lu]Lu-MAPEGDOX (orange line), MAPAD (blue line), and [^177^Lu]Lu-MAPAD (red line).

The survival rates of the mice are shown in Figure 24. In all cases, the mice were sacrificed due to exceeding the tumor volume (2 cm^3^) or dimensions (one dimension > 2 cm). More specifically, one mouse in the control group was sacrificed on day 6, two were sacrificed on day 9, and only one mouse was then left in this group, which was also sacrificed on day 17 according to the endpoint criteria mentioned earlier. From the “cold” MAPAD-administered mice, one mouse was sacrificed on day 8. One mouse on day 9 and one on day 13 were euthanized from the [^177^Lu]Lu-MAPEGDOX group. In the [^177^Lu]Lu-MAPAD-administered group, no mice were sacrificed, but there was only one mouse that was found dead (day 35), most probably not due to experiment-related reasons (it had tumor dimensions within the limits, no weight loss or signs of pain/discomfort).

When compared to that of the control group, the tumor growth arrest is pronounced. As shown in Figure 24, less than a week since the initiation of the therapeutic efficacy study, one mouse from the saline group already had to be sacrificed, and 3 days later, another two mice had to be sacrificed. There was only one mouse left in less than two weeks. Until that day, the mice in all the treated groups had a statistically significant difference when compared to the control group. More specifically, [^177^Lu]LuCl_3_ reduced the tumor size by 71.93%, [^177^Lu]Lu-MAPEGDOX reduced it by 79.10%, and a 74.35% tumor volume reduction was observed for [^177^Lu]Lu-MAPAD 17 d post administration. While on that day (day 17), the MAPAD-treated group showed the maximum tumor volume arrest when compared to that of the control group (82.47%), the response of the different groups followed a more dynamic route with the [^177^Lu]Lu-MAPAD group taking the lead from day 24 until the end of the study (day 50).

From the beginning of the study and for almost two weeks, the tumor growth increased at an almost steady rate for the treated groups. After that point, the different response among the groups started to be more obvious, while at day 24, the growth indexes from all the groups were very similar. After that time point, the tumors started to differentiate again among the treatment groups, with the most distinct difference on day 42, when the TGI for [^177^Lu]LuCl_3_ was 1.46 ± 1.15, 2.92 ± 1.76 for the [^177^Lu]Lu-MAPEGDOX-treated animals, 2.57 ± 1.77 for the MAPAD-treated animals, and only 0.76 ± 0.72 for the [^177^Lu]-MAPAD-treated animals. This means that at day 42, the [^177^Lu]Lu-MAPAD group was 1.92-, 3.84-, and 3.38-fold smaller when compared to the [^177^Lu]LuCl_3_, [^177^Lu]Lu-MAPEGDOX, and MAPAD groups, respectively. At the end of the study (day 50), the TGI of the [^177^Lu]LuCl_3_ was 1.63 ± 1.30, 2.12 ± 1.78 for the [^177^Lu]Lu-MAPEGDOX, 2.52 ± 1.41 for the MAPAD, and 1.10 ± 0.63 for the [^177^Lu]Lu-MAPAD.

It is also worth noting that the study was ended 50 days after the intratumoral injection due to programming and not due to tumor growth or the overall condition of the animals.

## 3. Discussion

### 3.1. Functionalization with DOX and Bevacizumab

The binding and entrapment efficiency of the antibody onto the alginate of the MAPEG NPs was high (41.84% and 35.97%, respectively). C. Tsoukalas et al. described the coating of iron oxide nanoparticles with DMSA and their consequent functionalization with bevacizumab at a concentration of C_BVCZ_/C_IONPs_ = 0.5 mg/mL/8 mmol/L [26]. It is also worth mentioning that the simultaneous conjugation of DOX and BVCZ has been reported to reduce the amount adsorbed onto magnetite/gold (Fe_3_O_4_/Au) by about 50% for DOX and 25% for BVCZ as compared to the values corresponding to independent adsorption [27]. In our study, a similar trend was noted, even though the percentage was slightly reduced (~7%) when DOX alone was linked to the NPs (88.99% vs. 82.67% for independent vs. co-adsorption, respectively).

The drug loading percentage accomplished in this work is four times higher than previously described percentages for the same nanoparticles (45.27 wt.% vs. 11 wt.%) [28]. The loading and entrapment efficiency of DOX into our nanosystem (45.27 wt.% and 82.70 wt.%, respectively) was distinct in comparison to that of similar nanosystems reported by G. Zoppellaro et al., whose study showed that the maximum loading ability of alginate-coated magnetic NPs was 26 ± 2 wt.% and there was a high entrapment efficiency ranging from 40% to 80% [29]. Previously, a slightly lower drug loading ability of MagP (PMAA-g-EGMA) NPs was determined at 22 ± 2 wt.% [30]. An even lower loading efficiency of DOX through electrostatic interactions of trimethoxysilylpropyl–ethylenediamine triacetic acid (EDT)-stabilized IONs was reported to be 5 ± 0.05% [31]. Finally, doxorubicin as well as epirubicin have been linked to SPIONs, previously stabilized with citric acid through amide bond reactions (EDC and NHS) [32]. In this work, the percentage of DOX successfully linked was about 11% according to the mass of the hybrid, thus much lower than the corresponding percentage in our work.

A thermogravimetric analysis was used mostly for the quantification of the polymeric content (alginate and PEG) of the NPs as well as for the estimation of the conjugated moieties. In the literature, unprocessed alginate and 2 kDa PEG indicate thermal decomposition peaks at 232.7 °C and 275.6 °C, respectively [33,34]. Therefore, since both alginate and PEG are conjugated onto the NPs, these are expected to appear on the thermograms in the range of 150–350 °C. Since the MAPEG NPs were functionalized with BVCZ and DOX, the same amount of alginate and PEG is expected to be present in both curves. Therefore, the weight loss observed at temperatures from 150 to 800 °C is attributed to the antibody and the drug. It has been shown with differential scanning calorimetry (DSC) that the pure drug has endotherm peaks at 195 and 355 °C [35]. However, when it is strongly associated with nanoparticles, thermal decomposition may occur at higher temperatures [35,36]. When calculating the BE and DL percentages that occurred from the HPLC analyses, if the mass of the whole nanosystem is considered (mass of NPs + mass of DOX + mass of BVCZ), then the respective percentages are ~28% and ~32%. This means a total of 60% of both substances is conjugated, which is in accordance with the TGA results (Figure 4).

Functionalization with BVCZ and DOX increased their perceived volume and thus their D_h_ rather than their actual size when interacting with the cells, due to the extensive presence of additional functional groups that may adsorb water molecules. It is important to underline the limitations of the DLS technique for the determination of the size of the nanoparticles. In general, this technique provides the hydrodynamic diameter of the NPs and gives only an indication of the size of the NPs [37,38]. Therefore, it must be used in combination with other techniques in order to accurately evaluate their size. The parent nanoplatform (MAPEG) has been previously characterized regarding its morphology and properties in previous studies [19,28,29]. High D_h_ values, as determined by DLS measurements, can be attributed to inter- and intra-molecular interactions [39]. Also, the MAPAD nanoparticles have fluorescent properties, which might interfere with the measurement procedure, thus leading to much higher D_h_ values. As shown previously, the ζ-potential of the NPs before pegylation was negative (−40 mV), due to the negatively charged groups of the magnetic core (Fe-O^−^) and the carboxylate groups (-COO^−^) of alginate [19]. Upon nanoparticle pegylation, the ζ-potential of MAPEG approached neutrality (−7 mV), due to the reduction in the number of free carboxylic acid groups in the alginate. For the same reason, upon functionalization with the antibody and the drug, ζ_p_ showed a further increase. It is also important to mention that BVCZ is a large molecule with a molecular weight of 149 kDa and contains, among other groups, amine groups that cause an increase in the zeta potential value (from −7 to 16.7 mV). Additionally, the isoelectric point (pI) of BVCZ is 8.3, meaning that below this value, at about a neutral pH (7.4), it is positively charged [40,41]. Furthermore, the increase in the mean hydrodynamic diameter is also attributed to the conjugation of the antibody, as due to the large Mw of BVCZ, it is possible that it is bound to multiple nanoparticles, causing agglomerates. This has been reported in the literature before, in which magnetic NPs of 101 nm exhibited an increase in D_h_ and ζ_p_ following functionalization with a monoclonal antibody [42].

### 3.2. DOX Release

Since both DOX and BVCZ were attached onto the NPs via covalent bonding, a very low release is expected. Covalent bonds are very stable and difficult to break without the use of strong acids or bases. Therefore, a mixture of proteases was used, and release experiments were performed with the direct incubation of the DOX-loaded nanoparticles in PB with different pHs [25].

The covalent binding of DOX to the nanoparticles can result in increased loading and a reduced initial burst release but these advantages are countered by potential reductions in the total release of the drug from the NPs. Regarding DOX release, neutral as well as more acidic pHs were examined because it is known that at a tumor site, acidic and hypoxic regions overlap [43]. In our case, due to the stable amide bond of DOX to the NPs, only a small amount of drug was released at physiological pH (7.4), as well as under more acidic conditions (a pH of 5.5). However, when pronase was added, the several of the nonspecific endo- and exoproteases it contains managed to cleave the bond and cause an augmented release of DOX (90.94 ± 3.40%) (Figure 6). Provided that similar DOX release rates will exist in vivo, this would provide a large enough time window to the nanoparticles to reach the tumor site before any significant drug leakage occurs. In the literature, there are numerous studies that use doxorubicin as a chemotherapeutic agent. In the case of DOX covalently conjugated via a pH-sensitive hydrazone bond to IONs, Norouzi et al. reported a 29% release in the first 2 h, while an additional 4% of the loaded DOX was released within 24 h [31]. The cumulative release was ~35% under acidic conditions. To our knowledge, there is only one hybrid system, the one reported by D. Nieciecka et al., that has DOX bound with an amide bond, as in the other studies, it is loaded through electrostatic interactions and the majority of the loaded drug is released within a few hours in a slightly acidic medium [24,28,30,31,32,44]. In this particular study, the loading of DOX onto the SPIONs took place using crosslinking agents, while an alternating magnetic field in two different media (at a pH of 5.8 and serum) was used for the release of the chemotherapeutic.

### 3.3. Radiolabeling with ^177^Lu

The stable binding of the radioisotope could be attributed to the fact that the positively charged radioisotope ^177^Lu^3+^ binds to the alginate corona and specifically to its negatively charged -COO^−^. As previously reported, the direct labeling of the ^177^Lu-labeled MAPEG NPs resulted in a very stable radionanoconjugate up to 5 d post radiolabeling with >90% intact [^177^Lu]Lu-MIONs [19].

For the radiolabeling of the functionalized nanoparticles (MAPAD), different radiolabeling conditions were applied. The presence of the antibody does not allow high incubation temperatures (>65 °C) as denaturation may occur [45,46,47]. Therefore, since the temperature was lower, we gave the system more time to efficiently conjugate the radioisotope. Indeed, that approach was effective, and after incubating the nanoparticles with the radioisotope overnight at 40 °C, the radiochemical yields were >90% (93.41 ± 2.59%).

### 3.4. In Vitro Cytotoxicity

The MTT assay showed no remarkable cellular toxicity effect after 3 days of treatment with MAPEG, in accordance to previous studies [28,29,44]. However, cell response to radiation is time- and activity-dependent and an increase in the cytotoxic effect can be observed by actively targeted systems [48,49,50].

As reported by M. Hein and S. Graver, treatment with bevacizumab does not appear to affect tumor proliferation [51]. The cell response of eight different cancer cell lines of four different types of cancer (non-small cell lung, colorectal, breast cancer, and renal cell carcinoma) reported low levels of apoptosis in all the cell lines investigated, which were not further enhanced by treatment with BVCZ up to 96 h. Additionally, BVCZ in concentrations up to 1 mg/mL does not have a cytotoxic effect against MDA-MB-231 cells [52].

### 3.5. Cellular Uptake

To explore the route of the intracellular localization of the NPs functionalized with the antibody, we followed two different in vitro imaging protocols: fluorescence microscopy due to the fluorescent abilities of DOX and the observation of MIONs via Prussian blue staining under optical microscopy. Bevacizumab is an mAb that binds to an extracellular VEGF-A protein and inhibits its interaction with VEGF receptors, inhibiting the angiogenesis process. However, several reports have described an intracellular VEGF pool, and an interesting strategy to intracellularly deliver bevacizumab has been reported [53].

A fluorescence imaging signal detected from the nuclei of the 4T1 cells verified the presence of DOX there, where it could eventually be released from the NPs and exert its toxic activity. Since the surface of most cells is negatively charged, antibodies need to be positively charged for efficient pinocytosis. Since BVCZ has an isoelectric point in the range of 8–9 (8.3) and the environmental pH is below the pI of the antibody (7.4), this could indicate that the antibodies are adequately taken up after administration [54,55,56]. These data strongly suggest that the whole nanoparticle (MAPAD) is able to be internalized by the cells. Part of the fluorescent signal coming from the cell nuclei in both MAPEGDOX and MAPAD is due to the released DOX. However, the fact that MAPAD-treated cells exhibit more intense fluorescent properties indicate the internalization of the antibody-conjugated and DOX-loaded NPs into the cell.

Prussian blue staining supports the different behavior of MAPAD and MAPEGDOX as 4T1 cells treated with the latter show that MAPEGDOX NPs are found in the cytoplasm or even outside the cells, most probably due to non-specific interactions between the NPs and the cells, or even with the surface of the wells. As already mentioned, it is generally agreed that positively charged NPs show higher uptake by cells when compared to neutral or negative ones, due to favorable electrostatic interactions with the negatively charged cell membrane. However, it has been reported that oxidative stress can lead to a significant decrease in negative charges on the cell surface and that they also contain some areas with cationic sites, allowing for the binding of the negatively charged nanoparticles and resulting in a clustering of the particles followed by subsequent endocytosis [57]. Consequently, there are numerous parameters that affect nanoparticle/cell interactions [58]. The fact that MAPEG NPs do not show any cellular uptake has been reported before by Sarigiannis et al., whose study found that negatively charged MagAlg NPs showed increased uptake on glioblastoma cells whereas their pegylated counterparts demonstrated the total absence of cellular uptake [28].

### 3.6. Ex Vivo Biodistribution

Although in vitro experiments have shown enhanced results for both MDA-MB-231 and 4T1 cells, which both have VEGF expression and have been used for VEGF-related experiments, we proceeded to animal studies due to the fact that 4T1 is a very fast-growing cancer cell line with high vascularization and tumors are formed quickly [59,60,61].

The ex vivo profile of the intravenous administration of the ^177^Lu-labeled nanosystems investigated shows uptake by the reticuloendothelial system (liver and spleen) rather than renal clearance (Figure 16), as they are significantly larger than the cutoff for renal filtration (10 nm) [62]. Indeed, our results are in accordance with the trend of ^177^Lu-labeled SPIONs and gold NPs that showed pronounced liver and spleen uptake after intravenous administration [63,64]. The bone uptake, which is attributed to the release of the radioisotope from the radionanoconjugate, appears to be similar to the one reported for the ^177^Lu-DOTA-neurotensin peptide at 7 d p.i. (~11% of the ID/g which increases to 29.45 ± 2.1% at 14 d p.i.), although a chelator (DOTA) was used, in contrast to our direct labeling of the NP [65]. In general, increases in the net positive charge of antibodies result in increased blood clearance and increased tissue retention with a shorter half-life [54,55,56]. However, observations can be conflicting regarding the correlation between antibody clearance and pI [66]. Fast blood clearance (0.81 ± 0.08% ID/g at 24 h) of BVCZ functionalized Fe_3_O_4_-^99m^Tc-labeled NPs was also reported by Tsoukalas et al. [26]. Furthermore, spleen uptake was similar (5.38 ± 0.73% vs. 9.08 ± 3.80% ID/g, 1 d p.i.) while accumulation in the liver in the case of [^177^Lu]Lu-MAPAD was higher at 24 h (16.10 ± 1.39%) when compared to that of Fe_3_O_4_-DMSA-SMCC-BCZM-^99m^Tc (7.81 ± 1.25%). Finally the lung uptake of the ^99m^Tc-labeled nanoconstructs 24 h post administration (~8%) was similar to the uptake reported for the [^177^Lu]Lu-MAPAD NPs at 1 d p.i. (6.28 ± 6.41%).

Our MAPAD-labeled NPs could not reach the tumor after i.v. or i.p. injection (Figure 16 and Figure 17), possibly due to the fact that generally tumors have highly heterogeneous blood vessel distributions and strong stromal barriers to drug delivery systems [67,68]. The results of comparisons between i.v. and i.t. administration have been reported before; in one study, [^225^Ac]^225^Ac-Au@TADOTAGA after local administration showed a tumor accumulation of 5.21 ± 1.26% IA/g after 12 d, whereas after i.v. injection, the peak was noted at 2 h p.i. (4.05% ± 0.34% IA/g), which decreased over time [69]. Furthermore, the i.v. and i.t. ex vivo biodistribution profile of AuNPs, radiolabeled with Gold-198 (^198^Au) and loaded with DOX, was evaluated in SKOV-3 xenografts [70]. Similarly to our results, i.t. administration led to a pronounced uptake in the tumor up to 48 h p.i. with other organs demonstrating an uptake < 21% of the total distributed activity. The intratumoral administration of 37 MBq (1.0 mCi) of ^177^Lu–EuDPA/SiO_2_–NH_2_ NPs was investigated versus the i.t. administration of 37 MBq (1.0 mCi) ^177^LuCl_3_ at 48 h p.i. [71]. Over 70% of the IA was found at the tumor with limited radioactivity in the other organs measured, whereas after i.t. injection with ^177^LuCl_3_, radioactivity was distributed in the tumor (~12% IA/g) as well as in the liver, spleen, and stomach/intestine (<15% of the ID/g).

As mentioned earlier, and in accordance to the European Medicines Agency and the Nuclear Science and Technology Organization, Lutetium chloride is a radiopharmaceutical precursor and is not intended for direct use in patients [72,73,74]. It is only to be used for the radiolabeling of carrier molecules that have been specifically developed and authorized for this purpose.

### 3.7. Therapeutic Efficacy

In our therapeutic efficacy study, we observed a slightly similar therapeutic response of the [^177^Lu]LuCl_3_ and the [^177^Lu]Lu-MAPAD groups at the end of the study (Figure 22). This could be attributed to the fact that [^177^Lu]LuCl_3_ was diffused in a more uniform manner inside the tumors and therefore caused more pronounced damage even though it did not remain in the tumor to same extent as the radiolabeled nanostructures [75]. Also, the fact that the pH at the tumor sites is slightly acidic or even close to neutral may cause the transformation of the [^177^Lu]LuCl_3_ into [^177^Lu]Lu(OH)_3_, leading to colloid formation and thus causing the prolonged retention of a fraction of the injected [^177^Lu]LuCl_3_ at the injection site. However, as previously mentioned, regulations clearly state that [^177^Lu]LuCl_3_ does not have any therapeutic application in clinical medicine and is not intended for use in patients. The presence of free [^177^Lu]LuCl_3_ will lead to increased bone marrow toxicity (osteosarcoma formation, myelosuppression), kidney damage, and hematopoietic stem cell damage [72,73,74].

Intratumoral administration is a very promising method that has been used for the local delivery of therapeutics. As we have seen from our previous work, Au NPs radiolabeled with the alpha emitter ^225^Ac showed significant tumor growth arrest after three intratumoral injections in glioblastoma (U87MG) xenografts up to 22 days [69]. Recently, K. Żelechowska-Matysiak et al. reported the enhanced therapeutic efficacy of intratumorally injected DOX-^198^AuNPs-Tmab over HER-2-overexpressing tumor-bearing mice. After 28d, a dose of 5 MBq (as in our study) of DOX-^198^AuNPs-Tmab reduced the tumor size by 77.5 ± 8.8%, while an 82.2 ± 8.5% tumor volume reduction was observed for the double radioactivity, in comparison to the tumor volume of the control group [70]. These results are comparable to those of our study, in which with the same amount of radioactivity, [^177^Lu]Lu-MAPAD caused a 74.35% tumor volume reduction at 17 d p.i. Similar results have also been reported in other studies that used ^198^Au-labeled AuNPs intratumorally injected in prostate-tumor-bearing mice [76,77,78]. The group of Jiang X. et al. evaluated the local injection of BVCZ against triple-negative breast cancer xenografts, causing a tumor inhibition rate of 32.8 ± 3% [79]. The anti-tumor efficacy of ^131^I-CC49-APTES@SPIONs was investigated for LS174T-bearing mice up to 14 d, which, after one intratumoral administration, showed a significant tumor retention when compared to the control groups [80]. The therapeutic potential of ^177^Lu-EuDPA/SiO_2_-NH_2_ NPs was evaluated after i.t. administration in HT-29 colon cancer xenografts with a significant therapeutic effect up to 17 d [71]. Another work that supports the efficacy of i.t. administration is that by the group of Yook et al., who used gold nanoseeds labeled with ^177^Lu (4.5 MBq) in mice with subcutaneous MDA-MB-468 tumors and monitored them for tumor growth up to 90 d or until the tumor size reached 12 mm [81].

It is of major importance to note that a therapeutic efficacy study using a triple-negative cancer cell line such as 4T1, which has an aggressive phenotype, may be more complicated [82]. It is possible that not all tumors develop in the same way as even a slight difference in the number of tumor cells inoculated can cause a burst augmentation of tumor size. Additionally, it is likely that at the initiation of treatment, large cell clusters (<1–2 mm^3^) do not form similar vasculatures, differentiating the diffusion of the radiopharmaceutical [83,84]. The very fast tumor progression is also supported by the results of a group of animals that received a saline injection (control group). In another study using 4T1 cells, the median overall survival of the control group was 8 d, and mice administered with single (9.25 or 18.5 MBq) or fractionated (2 × 9.25 or 18.5 + 9.25 MBq, administered on day 0 and day 10) doses of ^177^Lu-NM600 had a median survival of 21–26 d [85]. Therefore, the survival in our study was remarkable (50 d) when compared to the one reported by R. Hernandez et al., in which, although their mice received a much higher activity (2 × 9.25 MBq or 18.5 + 9.25 MBq) of the ^177^Lu-conjugate, the endpoint of the studied groups bearing these tumors was only 26 d. Furthermore, in our case, our survival rate was achieved with significantly lower doses (5 MBq), leading to less exposure to the bone marrow when compared to higher administered doses (9.25, 18.5, or 28 MBq) and levels of observed dose-dependent radiotoxicity. In another study, paclitaxel-loaded amphiphilic cyclodextrin NPs were administered in 4T1 tumor-bearing mice, and tumor monitoring was only performed for 14 days [86]. Pegylated liposomes were injected intravenously every other day and seven doses were administered; 24 h after the final dose, the mice were sacrificed [87].

## 4. Materials and Methods

^177^Lu isotope presents serious health threats and requires special radioprotective precautions during its handling to reduce the risk of harm. All radiolabeling procedures and work associated with radiolabeled compounds were conducted in a radiochemistry facility that has the necessary infrastructure, expertise, and licensing to safely conduct experiments with radioisotopes.

Water for injection was purchased from DEMO S.A. (Krioneri Attiki, Greece). All other reagents and solvents used in these studies were obtained from commercial sources without further purification. Water was deionized to 18 MΩ⋅cm using an Easypure water filtration system (Barnstead International, Dubuque, IA, USA). Doxorubicin hydrochloride was acquired in its commercial form Adriblastina^®^ (50 mg/25 mL, Pfizer, New York, NY, USA) and Bevacizumab was acquired as Avastin^®^ (100 mg/4 mL, Roche Genentech, San Francisco, CA, USA). Reverse-phase high-performance liquid chromatography (RP-HPLC) was used for the determination and quantification of doxorubicin and bevacizumab using an NUCLEOSIL 100-5 C18 (Macherey-Nagel, Dueren, Germany) and a TSKgel G3000SWxl (Tosoh Bioscience, Griesheim, Germany) column, respectively. The samples were shaken and heated using a Lab Companion CBS-350 heating shaker (JEIO TECH, Daejeon, Republic of Korea) and the samples were heated using a Digital Thermoblock TD 200 (Falc Instruments, Treviglio, Italy). Centrifugation was performed with a Hermle Z 326K centrifuge (Wehingen, Germany). For magnetic separation, a Nd-Fe-B magnet was used.

Lutetium-177 was purchased from POLATOM (Otwock, Poland). Radioactivity of [^177^Lu]LuCl_3_ and the radiolabeled nanoparticles was measured using a dose calibrator (Capintec, Ramsey, NJ, USA). Glass microfiber chromatography paper impregnated with silica gel (ITLC-SG) was purchased from Agilent Technologies (Santa Clara, CA, USA) and, along with a radio-TLC scanner (Scan-Ram, LabLogic, Sheffield, UK), was used in the determination of radiolabeling yield/purity during radiolabeling and stability studies. The mobile phase of the ITLC-SG analysis was 0.1 M citric acid. The percentage of ^177^Lu incorporated onto the NPs was calculated as 100 × (counts at application point/total counts). Data collection and analysis were performed with Laura software v. 5.0.4.29. Human serum was acquired from Sigma-Aldrich (St. Louis, MO, USA).

The triple-negative murine 4T1 breast cancer cell line mimicking stage IV human breast cancer, human adenocarcinoma MDA-MB-231, MCF7, and SKBR3 cell lines, and MDA-MB-231 transfected cells (M165) were used. All cell lines were acquired from the cell bank of the Laboratory of Radiobiology, Institute of Nuclear & Radiological Sciences & Technology, Energy & Safety, NCSR “Demokritos”. The cells were free of mycoplasma contamination, as judged visually under microscope observation and by regular 4′,6-diamidine-2′-phenylindole dihydro-chloride (DAPI) staining of the cell cultures.

The media for the cultures (Dulbecco’s Modified Eagle’s Medium (DMEM) and Roswell Park Memorial Institute 1640 Medium (RPMI)) were purchased from Biowest (Riverside, MO, USA), and the MTT reagent (3-[4,5-dimethylthiazol-2-yl]-2,5-diphenyl-tetrazolium bromide) was obtained from Applichem (Darmstad, Germany). All other reagents and solvents used were obtained from ThermoFisher Scientific (Waltham, MA, USA): fetal bovine serum (FBS), 100 U/mL penicillin, 0.1 mg/mL streptomycin, trypsin-EDTA solution (0.25% trypsin/0.53 mM EDTA), phosphate-buffered saline (PBS) at a pH of 7.4, dimethyl sulfoxide (DMSO), and 3-(4,5-dimethylthiazol-2-yl)-2,5-diphenyltetrazolium bromide (MTT). All procedures were performed in a laminar flow hood under sterile conditions to prevent contamination of cell cultures, while all the tools used in our study were cleaned with ethanol before use (70%). All cell lines were grown in DMEM, at a pH of 7.4, supplemented with 10% FBS, 100 U/mL of penicillin, 2 mM glutamine, and 100 μg/mL of streptomycin. The 4T1 cell line was grown in RPMI medium supplemented with 10% FBS, 100 U/mL of penicillin, 2 mM glutamine, and 100 μg/mL of streptomycin. Optical density measurements in the in vitro experiments were conducted using a LabSystems Multiskan RC microplate reader (Thermo Fisher Scientific, MA, USA). Fluorescence images were obtained with an M834FLR OMAX trinocular compound EPI-fluorescence microscope with 1.3 MP CMOS camera (green filters: excitation of 490–540 nm and emission of 590 nm).

For the animal experiments, SCID (severe combined immunodeficiency) mice of both genders were used. The mice were housed in individually ventilated cages (IVC) with constant temperature (22 ± 2 °C) and humidity (45–50%) and a 12 h light/dark cycle, with free access to food and water. Animals were obtained from the breeding facilities of the Institute of Biosciences and Applications, NCSR “Demokritos”. Our experimental animal facility is registered according to the Greek Presidential Decree 56/2013 (Reg. Number: EL 25 BIO 022), in accordance with European Directive 2010/63, which is in accordance with national legislation regarding the protection of animals used for scientific purposes. All applicable national guidelines for the care and use of animals were followed. The study protocol was approved by the Department of Agriculture and Veterinary Service of the Prefecture of Athens. These studies have been further approved by our institutional ethics committee, and the procedures followed are in accordance with institutional guidelines.

Murine 4T1 mammary carcinoma xenografts were developed for the biodistribution and therapeutic efficacy studies. Intravenous and intraperitoneal injections were performed using 1 mL BD insulin syringes (29G), whereas for the intratumoral injections, 0.3 mL BD Micro-Fine Plus insulin syringes (30G) were used. Animals were sacrificed using isoflurane 1000 mg/g (Iso-Vet, Chanelle Pharma, Loughrea, Ireland). The radioactivity of samples and syringes was measured using a dose calibrator (Capintec, Ramsey, NJ, USA). A Cobra II automatic gamma counter (Canberra, Packard, Schwadorf, Austria) was used to measure the radioactivity of each organ and blood sample in ex vivo biodistribution studies.

### 4.1. Synthesis of MAPAD

The functionalization of MAPEG with the antibody (BVCZ) and the chemotherapeutic drug (DOX) was performed using crosslinking agents and more specifically, 1-ethyl-3-(3-dimethylaminopropyl)carbodiimide hydrochloride (EDC) and N-hydroxysuccinimide (NHS). The first step was to change the solvent from water to 4-morpholinoethanesulfonic acid (MES) buffer (at pH of 5.5) which is more suitable for this reaction. To begin with, MAPEG (0.5 mg) was added to MES buffer, and the sample was centrifuged for 10 min at 10,000 rpm. Then the supernatant was removed, and EDC (0.17 mol) and NHS (0.39 mol) were dissolved in 200 μL MES buffer. The crosslinking agents were added to the sample to which MES buffer was also added to a final volume of 1 mL. The mixture was allowed to react for 30 min at 40 °C and 850 rpm. Afterwards, centrifugation for 10 min at 10,000 rpm was performed in order to remove the unreacted reagents, and the solvent was changed to phosphate buffer with a pH of 7.4. The drug and the antibody were added to the sample: 0.5 and 1 mg, respectively. The mixture was left to react overnight at 40 °C and 850 rpm, and after 24 h, the solvent and the unreacted substances (DOX and BVCZ) were removed by centrifugation.

In order to verify and quantify the successful functionalization of MAPEG with the antibody and the drug, high-performance liquid chromatography (HPLC) was used. At the end of the functionalization, samples were centrifuged in order to remove the unloaded BVCZ and DOX. The supernatant of this centrifugation was analyzed with HPLC, thus indirectly leading to the determination of the actual amount of BVCZ and DOX loaded on the NPs. Results were extracted based on a standard curve method and were plotted after analyzing control samples containing the initial amount of added BVCZ and DOX. By subtracting the amount of unloaded antibody and drug from the initial amounts of each used for the functionalization of MAPEG, it was possible to determine the exact amount of loaded substances.

Conjugation of the antibody was confirmed and quantified in isocratic solvent system (100% PBS buffer at pH of 7.4) using a TSKgel size exclusion column, while UV absorption was set at 280 nm. Reference samples of bevacizumab were taken and analyzed with HPLC. The quantity of the antibody conjugated for every functionalization was determined indirectly, i.e., after comparing the absorption of the unreacted BVCZ with the reference curve and thus calculating the BVCZ as shown in the following equations:

(5) $$\mathrm{Binding}\;\mathrm{Efficiency}\;(\%\;\mathrm{BE},\;\mathrm{wt}.\%)=\left(\frac{\mathrm{amount}\;\mathrm{of}\;\mathrm{bound}\;\mathrm{BVCZ}}{\mathrm{amount}\;\mathrm{of}\;\mathrm{NPs}\;+\;\mathrm{amount}\;\mathrm{of}\;\mathrm{bound}\;\mathrm{BVCZ}}\right)\;\times \;100\%$$

(6) $$\mathrm{Entrapment}\;\mathrm{Efficiency}\;(\%\;\mathrm{EE},\;\mathrm{wt}.\%)=\left(\frac{\mathrm{amount}\;\mathrm{of}\;\mathrm{bound}\;\mathrm{BVCZ}}{\mathrm{total}\;\mathrm{amount}\;\mathrm{of}\;\mathrm{added}\;\mathrm{BVCZ}}\right)\;\times \;100\%$$

For the determination of doxorubicin, reverse-phase high-performance liquid chromatography (RP-HPLC) was performed with a gradient elution solvent system consisting of acetonitrile (ACN)/0.1% trifluoroacetic acid (TFA) and H_2_O/0.1% TFA, and a C18 Nucleosil column (min 1–10: 100% H_2_O/TFA, min 10–25: 20% H_2_O/TFA and 80% ACN/TFA, min 25–26: 100% ACN/TFA, and min 26–30: 100% H_2_O/TFA) while absorption was set at 480 nm. The percentage of the loaded DOX is expressed by the following equations:

(7) $$\mathrm{Drug}\;\mathrm{Loading}\;(\%\;\mathrm{DL},\;\mathrm{wt}.\%)=\left(\frac{\mathrm{amount}\;\mathrm{of}\;\mathrm{loaded}\;\mathrm{DOX}}{\mathrm{amount}\;\mathrm{of}\;\mathrm{NPs}+\mathrm{amount}\;\mathrm{of}\;\mathrm{loaded}\;\mathrm{DOX}}\right)\;\times \;100\%$$

(8) $$\mathrm{Entrapment}\;\mathrm{Efficiency}\;(\%\;\mathrm{EE},\;\mathrm{wt}.\%)=\left(\frac{\mathrm{amount}\;\mathrm{of}\;\mathrm{loaded}\;\mathrm{DOX}}{\mathrm{total}\;\mathrm{amount}\;\mathrm{of}\;\mathrm{added}\;\mathrm{DOX}}\right)\;\times \;100\%$$

#### 4.1.1. Thermogravimetric Analysis

The degree of NP functionalization was also determined by thermogravimetric analysis (SDT-Q600, TA Instruments, New Castle, DE, USA) in a standard alumina pan heated from 30 °C to 800 °C with a nitrogen flow rate of 100 mL/min and a heating rate of 10 °C/min.

#### 4.1.2. Dynamic Light Scattering

The hydrodynamic diameter (D_h_) and polydispersity index of nanoparticles dispersed in deionized H_2_O were determined with a ZetaSizer Nano series Nano-ZS (Malvern Instruments Ltd., Malvern, UK) equipped with a He-Ne laser beam at a wavelength of 633 nm and a fixed backscattering angle of 173°. For the measurements, the materials were suspended in water, and the concentration of the colloid suspensions used for the measurement was 0.0125% *w*/*v* (g/100 mL) in Fe_2_O_3_. The zeta potential (ζ_p_) of the nanoparticles was measured with the same instrument as the average of 100 runs in the phase analysis light scattering (PALS) mode after equilibration at 25 °C.

### 4.2. DOX Release

The in vitro DOX release from MAPAD was studied in three different media: phosphate buffer (PB) at pH of 7.4, a more acidic PB (pH of 5.5), and another PB medium (pH of 5.5), which contained pronase at a concentration of 1 mg/mL [88]. Pronase is a commercially available enzymatic mixture of the extracellular fluid of Streptomyces griseus, and it contains numerous proteases and peptidases capable of hydrolyzing most peptide bonds [89]. The temperature was set to 37 °C, and samples were mildly shaken and protected from light. In all cases, 100 μL of the DOX-loaded NPs was dispersed in 1 mL medium and at predetermined time intervals (1, 2, 4, 12, 24, 48, and 72 h). NPs were isolated with magnetic separation, and the release medium was removed and replaced with fresh medium. Concentration of the released DOX was evaluated in the supernatant. Evaluation was performed via RP-HPLC at 480 nm.

### 4.3. Radiolabeling with ^177^Lu

**Figure 25:**
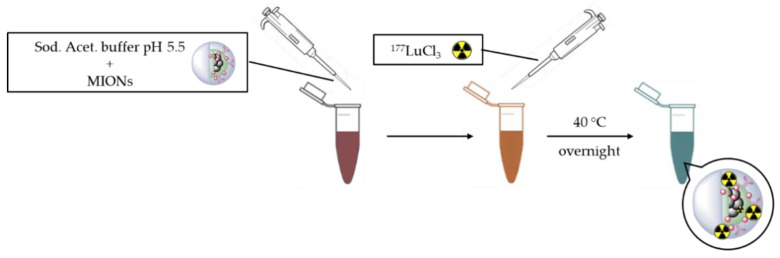
Reaction scheme of the radiolabeling procedure of MIONs with ^177^Lu.

Lutetium-177 was acquired in the form of [^177^Lu]LuCl_3_ in a 0.04 M HCl solution. Direct radiolabeling was achieved for both types of MIONs (MAPEG and MAPAD). Nanoparticles (50 μL, C_MAPAD_ = 0.5 mg/mL) were added to trace-free sodium acetate buffer (at pH of 5.5). Then, 10 to 50 MBq of [^177^Lu]LuCl_3_ was added, after which the mixture was slightly vortexed and consequently incubated at 40 °C overnight, resulting in our final ^177^Lu-labeled nanosystem as shown in Figure 25.

For radiochemical analysis, ITLC-SG (citric acid, 0.1 M) was used, in which [^177^Lu]Lu-MIONs remained at the application point (Rf = 0.0–0.2) while unbound ^177^Lu^3+^ was detected at the solvent front (Rf = 0.8–1.0). The radiolabeling yield and stability were estimated after integration of the peaks representing the radiolabeled NPs and unbound ^177^Lu, as demonstrated in Figure 7a in the results section.

In vitro stability studies of the [^177^Lu]Lu-MIONs at RT and serum were performed. Specifically, one part of the radiolabeled NPs was incubated with nine parts of human serum at 37 °C. In vitro stability was assessed up to 14 d post incubation and analyzed by ITLC-SG, using 0.1 M citric acid as the mobile phase. All experiments were performed in triplicate from three independent radiolabeling procedures.

### 4.4. In Vitro Cytotoxicity

In this study, five different breast cancer cell lines were used in order to evaluate the different response against cell lines with different levels of VEGF expression. Expression of the A isoform of VEGF (VEGF-A) is different in the various cancer cell lines, with the highest expression being observed in 4T1, MDA-MB-231, and M165 [59,60,90]. The in vitro cytotoxicity of the prepared MAPEG and MAPAD as well as the ^177^Lu-labeled MAPEG and MAPAD against the various cell lines was evaluated by a 3-(4,5-dimethylthiazol-2-yl)-2,5-diphenyltetrazolium bromide (MTT) colorimetric assay.

The cell cultures were maintained in flasks and were grown at 37 °C in a humidified atmosphere of 5% CO_2_ in air. The flasks were observed by means of optical microscopy to assess the degree of confluency. Subconfluent cells were detached using a 0.25% trypsin/0.53% mM ethylenediaminetetraacetic acid (EDTA) solution, while the subcultivation ratio for each cell line has been previously mentioned above. Once the cells had covered approximately 60–80% of the available area in the flask, the growth medium was removed, and the adherent cells were rinsed with PBS (~10 mL). In order to detach the cells from the surface of the flask, 2 mL of trypsin-EDTA solution was added, and the cells were incubated for a few minutes (depending on the cell line) in the incubator. Then, growth culture medium (10 mL) was added to inactivate the trypsin, followed by gentle pipetting to break any existing aggregates. For the determination of the number of the cells in a culture, a Neubauer chamber was used. To begin with, a drop of the cell suspension is carefully deposited between a cover glass and the counting chamber and is introduced into the grid under capillary action. Then, the number of cells in the chamber is determined by direct counting using optical microscopy. The appropriate number of cells were transferred to a 96-well plate and were allowed to grow overnight at 37 °C in a 5% CO_2_ incubator. For the 24 h protocol, 15 × 10^3^ cells were seeded per well, whereas for the 48 and 72 h protocols, 8 × 10^3^ and 4 × 10^3^ cells/well were seeded, respectively. The cells were treated with increasing concentrations of the complexes under investigation. The examined concentrations were 0.8125–13 μg[Fe_2_O_3_]/mL containing to 0.625–10 μg_DOX_/mL. Tandem experiments were performed with plain DOX and MAPEG at the same concentrations as MAPAD in order to enable a direct comparison. The ^177^Lu-labeled MIONs were evaluated with activities ranging from 0.3125 to 5 MBq/mL (corresponding to 0.8125–13 μg[Fe_2_O_3_]/mL and 0.625–10 μg_DOX_/mL). In every plate, some wells were filled again with fresh medium and used as our control. After the various incubation periods, medium was removed and replaced with 100 μL of MTT dissolved in the growth medium (1 mg/mL). After 4 h of incubation with the MTT, the latter was aspirated, and isopropanol (100 μL) was used to solubilize the formazan crystals. Absorbance was recorded at 540 nm, and results are expressed as noted from the following equation:

(9) $$\mathrm{Optical}\;\mathrm{Density}\;(\%\;\mathrm{of}\;\mathrm{control})=\frac{\mathrm{mean}\;\mathrm{optical}\;\mathrm{density}\;\mathrm{of}\;\mathrm{treated}\;\mathrm{cells}}{\mathrm{mean}\;\mathrm{optical}\;\mathrm{density}\;\mathrm{of}\;\mathrm{untreated}\;\mathrm{cells}}\;\times \;100\%$$

The final percentage of cell viability was expressed as the mean value ± standard deviation (MV ± SD) of three independent experiments.

### 4.5. Fluorescence Microscopy

The cellular uptake of samples was comparatively studied by fluorescence imaging in 4T1 cells. Cells were seeded in 24-well plates containing sterile coverslips and incubated for a period of 24 h at 37 °C and 5% CO_2_ to obtain approximately 80% confluence. Then, the culture medium was removed and replaced by culture medium containing each sample at a concentration of 10 μg_DOX_/mL. Control samples only with medium (without DOX) were also examined under the microscope to verify that there was no signal at the examined wavelength for any other reason other than the presence of DOX. After 1 and 4 h of incubation, the medium was removed, and the cells were washed with PBS and fixated with 4% paraformaldehyde in PBS for 20 min at 37 °C. Finally, the fixated cells were washed with PBS, and the coverslips were removed and placed on glass slides to be studied via fluorescence microscopy.

### 4.6. Prussian Blue

The visualization of NP uptake by 4T1 cells was achieved by the Prussian blue staining method. Cells were seeded in 12-well plates containing sterile coverslips at a cell density of 1 × 10^5^ cells per well and incubated for 24 h. The culture medium was then removed and replaced by culture medium containing each sample at a concentration of 13 μg_Fe_/mL (corresponding to 10 μg_DOX_/mL, same concentration as in the fluorescence imaging experiments). After 1 and 4 h of incubation, the medium was removed, and cells were washed with PBS and fixated with 4% paraformaldehyde in PBS for 20 min at 37 °C. The fixated cells were washed twice with PBS, incubated with Perls solution (1:1 solution of 4% *w*/*v* K_4_Fe(CN)_6_·3H_2_O in PBS and 4 M HCl in PBS) for 30 min, and washed again with PBS. Nuclear Fast Red 0.02% solution was added for 5 min and quickly washed with PBS. The coverslips were removed and placed on glass slides to be studied via optical microscopy.

### 4.7. Ex Vivo Biodistribution

All animal studies were performed on 6–8-week-old mice. SCID mice weighing 15–25 g were used, and each mouse was subcutaneously inoculated with a 4T1 cell suspension in growth medium (100 μL). Each injection contained approximately 10^7^ cells and was performed in the region under the left front limb. Approximately one week after cell inoculation, the tumor was of a palpable size (~200 mm^3^) appropriate for experimentation.

Administration of the ^177^Lu-labeled radioconjugates was performed in three different ways: intravenously (i.v.), intraperitoneally (i.p.), and intratumorally (i.t.). In this case, calculations were performed according to the amount of loaded DOX. Since it is not appropriate to inject large volumes during i.t. administrations, 60 μL of radiotracer was injected for all methods of administration (i.t., i.v., and i.p.).

Animals were euthanized at specific time points (*n* = 3 animals per time point) in a chamber saturated with isofluorane vapors, and the organs and tissues of interest (i.e., heart, liver, spleen, lungs, kidneys, stomach, intestines, pancreas, and bones), the tumor, and samples of blood, muscles, and urine were excised, weighed, and measured in an automatic γ-counter.

The injected activity (IA) in each mouse was estimated by measuring the radioactivity of the syringe before and after injection with a dose calibrator and thereafter subtracting the remaining radioactivity in the tail, as well as the background counts during the measurement. The measurements were expressed in counts per minute (cpm). Finally, the accumulation of the radiolabeled MIONs in organs and tissues at each time point was expressed as the mean percentage of injected activity per gram ± standard deviation (% IA/g ± SD), using an appropriate sample as a standard. In each experiment, three standard samples were made, each corresponding to 10% of the total injected dose, according to the following equation:

(10) $$\%\;\mathrm{IA}/g=\frac{\mathrm{cpm}\;\mathrm{of}\;\mathrm{organ}}{\mathrm{weight}\;\mathrm{of}\;\mathrm{organ}\;\times \;\mathrm{cpm}\;\mathrm{of}\;\mathrm{IA}}\;\times \;100\%$$

Approximately 1 ΜBq of [^177^Lu]Lu-MAPAD, [^177^Lu]Lu-MAPEG, and [^177^Lu]LuCl_3_ per mouse was administered. All mice were euthanized 1, 2, and 7 d post injection, the organs/tissues were excised, and the % IA/g was calculated as mentioned above.

### 4.8. Therapeutic Efficacy

Therapeutic efficacy studies were performed in SCID mice bearing subcutaneous breast cancer tumors when the tumor reached a volume of about 200 mm^3^ (approximately 7 d after inoculation of 4T1 cells). Mice were randomly divided into five groups (*n* = 4 mice per group) and received one intratumoral injection of either normal saline (control group, 60 μL saline) or functionalized NPs’ MAPAD (60 μL/5 μg_DOX_/mL). The other three groups included the animals treated with the ^177^Lu-labeled conjugates: [^177^Lu]LuCl_3_ (60 μL/5 MBq), ^177^Lu-labeled NPs functionalized with DOX ([^177^Lu]^177^Lu-MAPEGDOX, 60 μL/5 MBq/5 μg_DOX_/mL), and ^177^Lu-labeled NPs functionalized with both DOX and BVCZ ([^177^Lu]^177^Lu-MAPAD, 60 μL/5 MBq/5 μg_DOX_/mL). Mice were monitored by measuring their body mass and potential signs of pain or unease three times a week. Tumor volume was monitored for 50 days using calipers and was calculated using the formula (length × width^2^)/^2^ [81]. The tumor growth index (TGI) for all animal groups was calculated by dividing the tumor volume at each measurement by the initial tumor volume at the initiation of the experiment (day 0), just before the intratumoral injection of ^177^Lu-labeled complexes. TGI was plotted vs. treatment time post injection.

### 4.9. Statistical Analysis

The data are presented as means ± standard deviations (SDs). For the in vitro studies and biodistribution studies, data were compared using an unpaired *t*-test with a significance level of *p* < 0.05. Asterisks indicate the statistical significance of the difference between the results (* *p* < 0.05, ** *p* < 0.01, *** *p* < 0.001). Absence of asterisks denotes a non-significant statistical difference. Analyses were performed using GraphPad Prism (San Diego, CA, USA) software (https://www.graphpad.com/quickcalcs/ttest1/?format=SD).

## 5. Conclusions

The iron oxide nanoparticles investigated in the present study, coated with sodium alginate and PEG, were effectively functionalized with doxorubicin and bevacizumab via covalent bonds. Direct radiolabeling with the therapeutic radioisotope Lutetium-177 (^177^Lu) was accomplished with yields >90%. The radiolabeled nanoconstructs had a high in vitro stability at room temperature and a moderate serum stability up to 14 d post radiolabeling. The in vitro cytotoxic profile of the MIONs (MAPEG, MAPAD) was investigated against five different breast cancer cell lines which have different expressions of VEGF. Our MTT assay did not show any remarkable level of MAPEG toxicity up to 72 h after treatment. The in vitro cytotoxicity of the ^177^Lu-labeled-MAPEG and MAPAD showed a radioactivity dose- and time-dependent decrease in viability. Fluorescence microscopy and Prussian blue staining were used in order to investigate whether the presence of the antibody caused any difference in the binding affinity or internalization capacity of the functionalized NPs. Indeed, BVCZ-loaded NPs (MAPAD) seemed to be effectively internalized in 4T1 cells even at 1 h of incubation. They also indicated uptake in the cell nuclei, while at the same time, cells incubated with MAPEGDOX (nanoparticles loaded with DOX without BVCZ) mostly remained in the cytoplasm of the cells. The ex vivo biodistribution up to 7 d post administration was in tumor-bearing mice with three different ways of administration. The intratumoral administration exhibited a highly localized and specific accumulation at the tumor site up to 7 d p.i. The therapeutic efficacy study after one intratumoral dose established the enhanced therapeutic capacity of our functionalized [^177^Lu]Lu-MAPAD NPs in 4T1 tumor-bearing mice.

Consequently, the overall in vitro and in vivo findings of this study strongly indicate that [^177^Lu]Lu-MAPAD nanoparticles have great potential as nanobrachytherapy agents against breast cancer.

## Data Availability

The data presented in this study are available in article.
